# Manganese Dioxide Nanoparticles Protect PC12 Cells Against H_2_O_2_-Induced Oxidative Stress Injury by Regulating PI3K/Akt-Mediated Autophagy

**DOI:** 10.3390/pharmaceutics18091156

**Published:** 2026-09-15

**Authors:** Weijian Zeng, Duanyang Zhou, Tianlong Wang, Zhan-Lu Ma-Högemeier, Song Cai, Bingfeng Liu, Chao Song, Ling Guo, Rihong Zhai, Xun Song, Zhendan He, Yun Dong

**Affiliations:** 1 College of Pharmacy, Shenzhen Technology University, Shenzhen 518118, China; zwj040820@163.com (W.Z.); wangtianlong@sztu.edu.cn (T.W.); mazhanlu@sztu.edu.cn (Z.-L.M.-H.); songxun@sztu.edu.cn (X.S.); hezhendan@sztu.edu.cn (Z.H.); 2 School of Pharmaceutical Sciences, Health Science Center, Shenzhen University, Shenzhen 518055, China; 2070245005@email.szu.edu.cn (D.Z.); caisong@szu.edu.cn (S.C.); rzhai@szu.edu.cn (R.Z.); 3 The Brain Cognition and Brain Disease Institute, Shenzhen Institute of Advanced Technology, Chinese Academy of Sciences, Shenzhen 518055, China; bf.liu@siat.ac.cn; 4 Anning First People’s Hospital Affiliated to Kunming University of Science and Technology, Kunming 650302, China; chaoge6870@163.com (C.S.); h0726136@kust.edu.cn (L.G.)

**Keywords:** oxidative damage, BSA-MnO_2_ NPs, neuroprotection, autophagy, PI3K/Akt signaling pathway

## Abstract

**Background:** Oxidative stress-mediated neuronal injury is critically involved in the pathogenesis of neurodegenerative disorders, including Alzheimer’s disease and Parkinson’s disease. Manganese dioxide (MnO_2_), owing to its intrinsic reactive oxygen species (ROS)-scavenging capacity, has emerged as a promising neuroprotective candidate; however, the underlying molecular mechanisms remain insufficiently defined. **Methods:** Bovine serum albumin-templated MnO_2_ nanoparticles (BSA-MnO_2_ NPs) were synthesized, and their protective effects were evaluated in H_2_O_2_-treated PC12 cells. **Results:** BSA-MnO_2_ NPs significantly inhibited H_2_O_2_-induced reductions in cell viability, ROS overproduction, and mitochondrial membrane potential disruption. Mechanistically, H_2_O_2_ increased both LC3-II and p62 levels, indicating impaired autophagic flux. Activation of autophagy by serum starvation alleviated H_2_O_2_-induced injury, whereas chloroquine exacerbated cellular damage and abolished the protective effects of BSA-MnO_2_ NPs, suggesting that the restoration of autophagy contributes to BSA-MnO_2_ NPs-mediated neuroprotection. Further analysis showed that BSA-MnO_2_ NPs enhanced Akt phosphorylation, while LY294002, a PI3K inhibitor, suppressed Akt activation, disrupted autophagy regulation, and eliminated their neuroprotective effects. In contrast, chloroquine did not affect Akt phosphorylation, indicating that PI3K/Akt signaling acts upstream of autophagy regulation. **Conclusions:** Collectively, these findings demonstrate that BSA-MnO_2_ NPs protect PC12 cells against H_2_O_2_-induced oxidative injury by restoring autophagy through the PI3K/Akt signaling pathway, highlighting a potential role of BSA-MnO_2_ NPs in the treatment of oxidative-stress-related neurodegenerative disorders.

## 1. Introduction

Oxidative stress, caused by excessive ROS, can damage neuronal cell membranes, proteins, and DNA, which thereby disrupts the cellular functions and eventually results in neurodegeneration [1,2]. For instance, oxidative stress-induced damage was found to lead to the aggregation of amyloid β (Aβ) proteins in Alzheimer’s disease and induced the degeneration of dopaminergic neurons in Parkinson’s disease [3,4,5]. Reducing oxidative stress-mediated neuronal damage remains a major challenge in neurodegenerative disorders.

Hydrogen peroxide (H_2_O_2_), an inducer of ROS overgeneration, is a primary contributor to oxidative stress damage and subsequent neuronal cell damage [6,7]. Accordingly, H_2_O_2_ is widely used as a neurotoxic agent to establish in vitro oxidative stress models in various neuronal cells lines. Evidence has shown that H_2_O_2_ causes excessive intracellular ROS production, mitochondrial membrane potential depolarization and decreased superoxide dismutase activity, which then induces caspase-dependent apoptosis in PC12 cells [8] and SH-SY5Y cells [9]. In HT-22 cells, H_2_O_2_ leads to the opening of the mitochondrial permeability transition pores and exacerbates mitochondrial dysfunction [10]. Additionally, H_2_O_2_ exposure induces autophagic death in stem cells [11] and lipid peroxidation in PC12 cells [12]. It has been proven that excessive ROS scavenging could effectively decelerate the progression of neuronal death [13]. Therefore, it is of importance to explore neuroprotective mechanisms to prevent oxidative stress damage.

MnO_2_ nanoparticles possess the ability to consume ROS, generate O_2_, and reverse the proinflammatory microenvironment, suggesting that MnO_2_ could be developed into a potential treatment for some diseases, such as cancers and neurodegeneration. For instance, multifunctional albumin–MnO_2_ could attenuate hypoxia and induce cancer cell death [14]. The combination of polyethylene glycol and MnO_2_ was found to protect cartilage against oxidative-stress-induced inflammation [15]. A MnO_2_-nanoparticle-dotted hydrogel has been shown to promote spinal cord repair via alleviating the ROS microenvironment and thereby improving the viability of mesenchymal stem cells [16]. Furthermore, a study has shown that macrophage-disguised FIY-loaded MnO_2_ nanoparticles efficiently consumed excessive ROS and attenuated proinflammation, and thereby were neuroprotective against ischemic stroke [17], whereas evidence has shown that nanosized Mn particles could be toxic to neuronal cells [18]. These studies indicate that MnO_2_ might exert opposite roles with respect to neurodegeneration and neuroprotection. Therefore, the application of MnO_2_ in neurodegenerative disorders remains largely unknown. Bovine serum albumin (BSA), an endogenous plasma protein, could enable nanoparticle transmembrane transport across the tight blood–brain barrier via targeting albumin receptors [19]. Moreover, BSA coating could preserve the catalytic sites of MnO_2_ nanoparticles, and its improved dispersion could also facilitate the exposure of additional catalytic-active sites [20]. Therefore, BSA-coated MnO_2_ nanoparticles could possess stronger antioxidant capacity than their aggregated bare counterparts in the context of neuronal oxidative damage. Herein, BSA-templated MnO_2_ nanoparticles (BSA-MnO_2_ NPs) were engineered, and their neuroprotective effects were systematically investigated in PC12 cells exposed to H_2_O_2_.

In this study, BSA-MnO_2_ NPs effectively rescued PC12 cells from H_2_O_2_-induced oxidative damage via activating the PI3K/Akt signaling pathway to restore autophagy. This study provides a novel insight into the neuroprotective potential of BSA-MnO_2_ against oxidative neuronal damage, which may facilitate the development of efficient therapeutic agents for oxidative stress-related neurodegenerative diseases.

## 2. Materials and Methods

### 2.1. Reagents

Dulbecco’s modified Eagle’s medium (DMEM) and phosphate buffered saline (PBS) were purchased from HyClone (Logan, UT, USA). Fetal bovine serum (FBS), penicillin/streptomycin and trypsin were obtained from Gibco (Thornton, Australia). The ROS assay kit (DCFH-DA, S0033S), the mitochondrial membrane potential assay kit (JC-1 kit, C2006), the Cell Counting Kit-8 (CCK-8, C0039), RIPA buffer, phenylmethanesulfony fluoride, and DAPI were purchased from Beyotime, Shanghai, China. Protease inhibitors and Phosphatase inhibitors 1 and 2 were purchased from Roche, Mannheim, Germany. Nuclease was purchased from Biotech, Shanghai, China. Pierce BCA Protein Assay kit was obtained from Thermo Fisher Scientific (Waltham, MA, USA). PVDF membranes was purchased from Bio-Rad (Hercules, CA, USA). The antibodies of LC3II (85306S), p62 (5114T), p-Akt, Akt, GAPDH, and horseradish peroxidase (HRP)-conjugated goat anti-rabbit antibody were purchased from Cell Signaling Technology (Danvers, MA, USA). Chemiluminescence ECL was purchased from MilliporeSigma (Burlington, MA, USA). Chloroquine (CQ), and LY294002 were obtained from MedChemExpress LLC (Monmouth Junction, NJ, USA). H_2_O_2_, MnCl_2_, and bovine serum albumin (BSA) were obtained from Sigma-Aldrich (St. Louis, MO, USA).

### 2.2. Preparation of BSA-MnO_2_

The preparation of BSA-MnO_2_ by directly mixing the solution of MnCl_2_ and BSA has been described previously [21]. Briefly, 100 mg of BSA was dissolved in 10 mL of distilled water. Then, 500 μL MnCl_2_ solution with a concentration of 12.26 mg/mL was added into the above BSA solution, and the mixed solution was kept stirring for 30 min at 600 rpm at room temperature. After stirring at 800 rpm for 2 h at 37 °C, the mixed solution was adjusted with 1 M NaOH to pH 11.0. Next, the solution was kept stirring at 800 rpm for 2 h at 37 °C and then dialyzed for 48 h. The obtained MnO_2_-BSA solution was stored at 4 °C.

### 2.3. Characterizations

The morphology of BSA-MnO_2_ was characterized using transmission electron microscopy (TEM, FEI TECNAI F30, Hillsboro, OR, USA). The size and the zeta potential of the nanoparticles were respectively measured using a Malvern Zetasizer ZS90 (Worcestershire, UK) and a ZatePALS Zeta Potential Analyzer (Brookhaven Instruments Corporation, New York, NY, USA). The surface composition and chemical state of Mn were measured using an XSAM800 X-ray photoelectron spectrometer (Kratos Analytical Ltd., Manchester, UK).

### 2.4. Measurement of O_2_ Production Catalyzed by MnO_2_

The O_2_ generation by prepared MnO_2_ was determined using a JPSJ-605F portable dissolved oxygen meter (Leici Instrument Co., Ltd., Shanghai, China). Briefly, BSA-MnO_2_ at different concentrations (0, 200, 400, and 800 μg/mL) in PBS was filled with argon, sealed with parafilm, and incubated with 1 mM H_2_O_2_ for 10 min. Subsequently, the dissolved O_2_ content in the solutions was recorded within 2 min.

### 2.5. Cell Culture and Drug Treatment Protocol

PC12 cells purchased from Pricella (Wuhan, China) were cultured in DMEM with 10% FBS, 100 U/mL penicillin and 100 U/mL streptomycin in a humidified atmosphere with 5% CO_2_ at 37 °C. PC12 cells were seeded into 96-well plates at a density of 2.5 × 10^4^ cells/well and into 6-well plates at a density of 2.5 × 10^5^ cells/well for 24 h, respectively. Cells were pretreated with different concentrations of BSA-MnO_2_ NPs for 24 h. After rinsing, H_2_O_2_ or other reagents were added for further incubation. After 24 h, the cells were used for a variety of experiments.

### 2.6. Cell Viability

Cell viability was measured using the CCK-8 assay kit. After PC12 cells in 96-well plates were treated with the application of various compounds for 24 h, 10 μL CCK-8 solution was added to each well and incubated at 37 °C for 30 min. Thereafter, the absorbance at 450 nm was determined using a microplate reader (BioTek, Winooski, VT, USA).

### 2.7. Measurement of Intracellular ROS

The ROS level was measured with the probe 2′,7′-dichlorodihydrofluorescein diacetate (DCFH-DA). Briefly, the cells seeded in 6-well plates were incubated in serum-free DMEM with 10 μM DCFH-DA at 37 °C for 30 min. Fluorescent images were captured in six different areas of each well under a fluorescent microscope (AxioVert A1, Zeiss, Germany), and the fluorescence intensity was measured using ImageJ V1.53t software. The percentage of ROS-positive cells was calculated as the total number of ROS-positive cells in each group/those in the control group × 100%.

### 2.8. Measurement of Mitochondrial Membrane Potential

The level of mitochondrial membrane potential was detected by a commercial cyanine JC-1 assay kit as described previously [13]. J-aggregates at higher concentrations appear red fluorescent in mitochondria, reflecting higher membrane potential, whereas J-monomers at lower concentrations appear green fluorescent, indicating lost membrane potential. Therefore, the fluctuation of mitochondrial membrane potential is expressed as the ratio of J-aggregate/J-monomer (red/green) fluorescence intensity. Briefly, the treated cells were incubated with 10 μM JC-1 solution at 37 °C for 30 min. Subsequently, images were captured in six different areas of each well under the fluorescence microscope. The changes in mitochondrial membrane potential were analyzed using ImageJ V1.53t software, represented by the red/green fluorescence intensity ratio.

### 2.9. Immunofluorescence

The treated cells of crawling slices were fixed with 4% paraformaldehyde for 20 min, and rinsed twice. Thereafter, the cells were permeabilized with 0.3% Triton X-100 and blocked with 5% BSA at room temperature for 1 h. After that, cells were incubated with LC3II antibody or SQSTM1/p62 antibody (1:500) in blocking solution at 4 °C overnight. Then, the cells were incubated with secondary antibodies, either FITC conjugated anti-mouse IgG or with FITC conjugated anti-rabbit IgG (1:2000) at room temperature for 2 h. Subsequently, cells were incubated with DAPI (1:1000) at room temperature in dark for 5 min, and the slices were mounted. The immunohistochemistry slices were observed under an inverted fluorescence microscope. The cells were photographed in six different areas of each well under fluorescent microscope. Images were analyzed using ImageJ V1.53t software. The percentage of each group was calculated as LC3II-positive or p62-positive cell number/total cell number × 100%.

### 2.10. Western Blot Analysis

All treated cells were washed twice with cold PBS and then lysed in RIPA buffer containing phenylmethanesulfony fluoride (1:100), protease inhibitors (1:50), Phosphatase inhibitors 1 and 2 (1:100) and nuclease (1:1000) on ice. The collected cell lysates were centrifuged at 12,000 rpm for 10 min at 4 °C, and the supernatant was collected. The total protein concentrations were quantified with a Pierce BCA Protein Assay kit. An equal amount of total protein (15 μg) of each group was separated on 10% or 15% SDS-PAGE gels and transferred onto PVDF membranes. Subsequently, the membranes were blocked with 5% milk in Tris-buffered saline containing 0.05% (*v*/*v*) Tween 20 (TBS-T) at room temperature for 1 h and then incubated with a primary antibody at 4 °C overnight. The antibodies were in different dilutions, as follows: p-Akt, Akt, LC3II, p62, GPADH in 1:1000 dilution. Afterwards, membranes were incubated with an HRP-conjugated secondary antibody at 1:5000 dilution for 1 h at room temperature. Reactive bands were visualized by the quantity one automatic imaging analysis system (Bio-Rad, Hercules, CA, USA) using enhanced chemiluminescence ECL. The grayscale of protein bands was calculated using ImageJ V1.53t software. The quantitative data obtained from ImageJ V1.53t software were processed. Target-protein expression was normalized against the grayscale value of GAPDH from the same lane. The normalized relative protein expression was calculated as the ratio of the integrated optical density of the target protein to that of GAPDH. Relative expression fold changes of each group were calculated with the control group as the baseline for semi-quantitative protein analysis. All experiments were performed in triplicate, and mean values were reported as final results.

### 2.11. Statistical Analysis

All the experiments were repeated at least three times. One-way analysis of variance (ANOVA) followed by a post hoc multiple-comparison Tukey test was used to analyze the differences among groups with GraphPad 11.0 software. All quantified data were expressed as means ± standard error of the mean (SEM). Mean values of * *p* < 0.05, ** *p* < 0.01, and *** *p* < 0.001 were considered to be significant.

## 3. Results

### 3.1. H_2_O_2_ Induces Oxidative Damage in PC12 Cells

**Figure 1:**
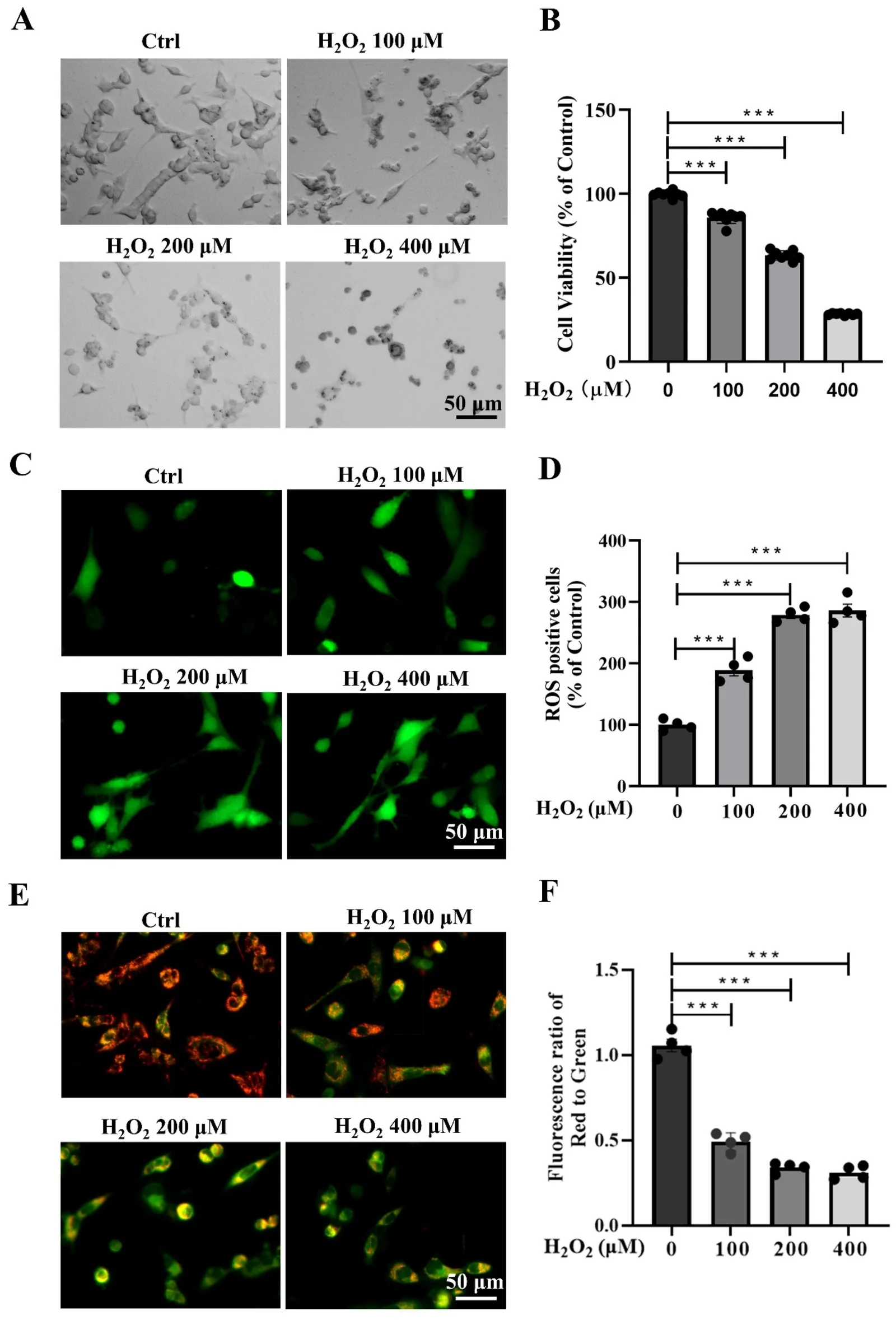
H_2_O_2_ induces oxidative stress damage in PC12 cells. Cultured PC12 cells were exposed to different concentrations (100, 200, 400 μM) of H_2_O_2_ for 24 h; the control group (Ctrl) was treated with PBS. (**A**) Representative images of the cell morphology. Scale bar = 50 μm. (**B**) Cell viability was detected using CCK-8 kit. (**C**) Treated PC12 cells were stained with DCFH-DA. 6-well plates with cells were observed under a fluorescence microscope. Scale bar = 50 μm. (**D**) Fluorescence density mean of ROS was calibrated. (**E**) Treated PC12 cells were stained with JC-1 kit. Scale bar = 50 μm. (**F**) Ratios of JC-1 red/green fluorescence optical density were analyzed by ImageJ software. All data in bar charts represent mean ± SEM from at least three independent experiments. *** *p*  <  0.001 versus the control group.

To study oxidative stress, PC12 cells were exposed to various concentrations (100, 200, and 400 μM) of H_2_O_2_ for 24 h. The cell viability was detected using CCK-8 kit. Our results showed that increased H_2_O_2_ concentrations caused a dose-dependent decrease in cell viability as compared to the control group (Figure 1A,B). H_2_O_2_ at 200 μM significantly decreased cell viability of PC12 cells (*p* < 0.01). To further investigate the vulnerability of PC12 cells to H_2_O_2_, ROS production was measured. Our data showed that 100 μM H_2_O_2_ markedly elevated ROS levels in PC12 cells (Figure 1C,D). Furthermore, mitochondrial membrane potential of PC12 cells was evaluated via JC-1 staining. As illustrated in Figure 1E,F, H_2_O_2_ also led to a significant dose-dependent decrease in the red/green ratio in PC12 cells, suggesting that the mitochondrial membrane potential was damaged. These results demonstrate that H_2_O_2_ induces oxidative damage in PC12 cells.

### 3.2. H_2_O_2_-Induced Oxidative Damage Is Associated with Autophagy Dysregulation

**Figure 2:**
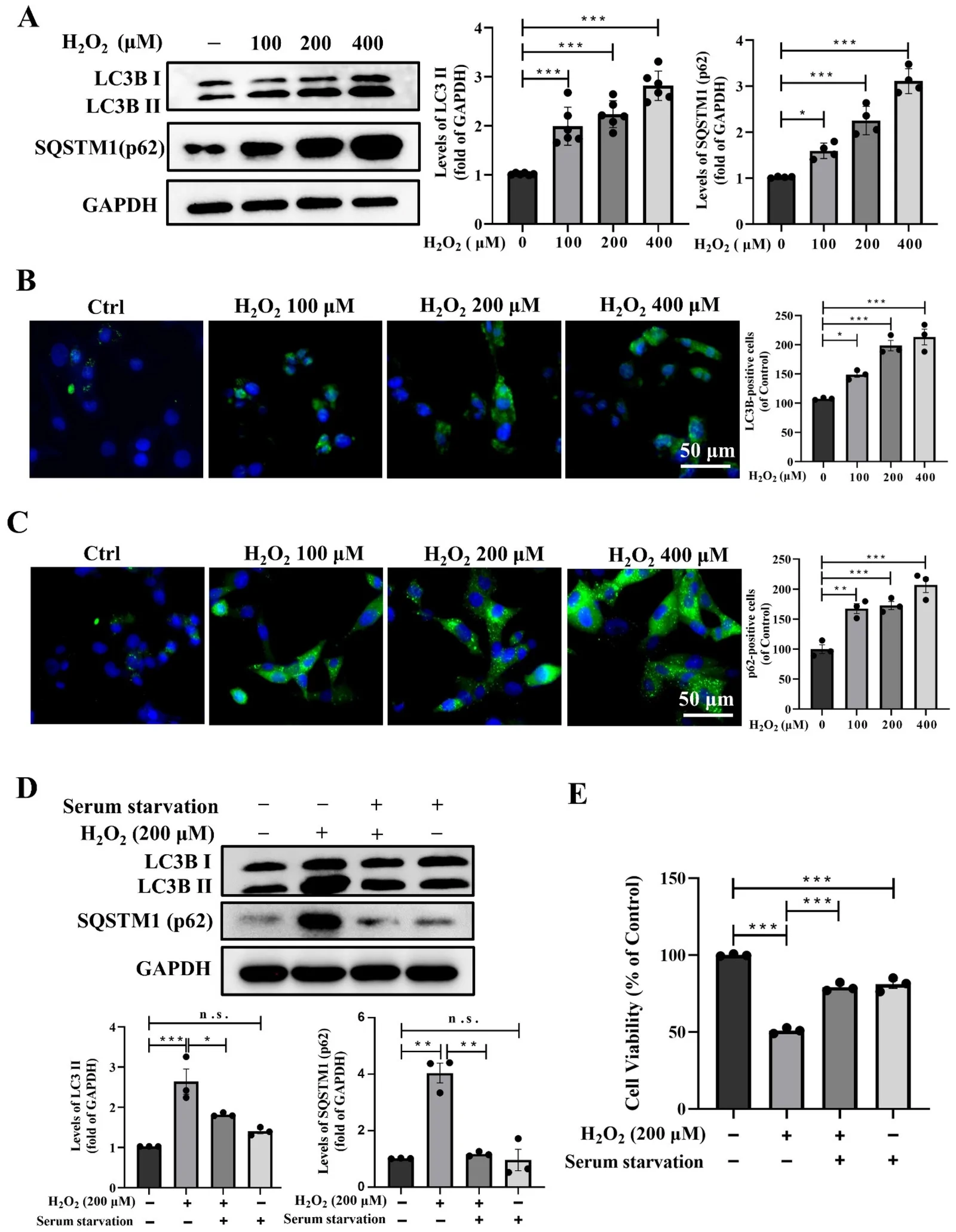
H_2_O_2_-induced oxidative damage is associated with autophagy dysfunction. (**A**) The protein expression levels of LC3 and p62 were determined by Western blot in PC12 cells treated with different concentrations of H_2_O_2_ (0, 100, 200, 400 μM) for 24 h, and expression of GAPDH served as loading control. The quantitation of LC3II and SQSTM1/p62 (p62) expression levels was calibrated. (**B**) LC3II was detected using immunocytochemistry and photographed under a fluorescent microscope, and images were analyzed. Scale bar = 50 μm. (**C**) p62 was detected using immunocytochemistry and photographed under a fluorescent microscope, and images were analyzed. Scale bar = 50 μm. (**D**) The protein expression levels of LC3 and p62 were determined by Western blot, and expression of GAPDH served as loading control. Quantitation of LC3II and p62 expression levels was calibrated. (**E**) Cell viability was detected. All data in bar charts represent mean ± SEM from at least three independent experiments. * *p*  <  0.05, ** *p*  <  0.01, *** *p* <  0.001 versus the H_2_O_2_ group or the control group (Ctrl). n.s., no significance.

Evidence has shown that oxidative stress is involved in autophagy dysfunction in neurodegenerative diseases [22]. To address whether autophagy was involved in oxidative damage in PC12 cells, LC3II and SQSTM1/p62 (p62) expression, which is widely used to monitor autophagy functions [21,22], was analyzed. As shown in Figure 2A, H_2_O_2_ dose-dependently increased LC3II levels and p62 levels, indicating that H_2_O_2_ suppressed autophagy flux and led to autophagy dysfunction. Similarly, the immunofluorescence results also showed H_2_O_2_ stimulation increased LC3II levels and p62 levels (Figure 2B,C). To further confirm that H_2_O_2_ caused autophagy dysfunction, serum starvation was applied to stimulate autophagy. PC12 cells stimulated by serum starvation for 4 h were incubated with 200 μM H_2_O_2_ for 24 h. Western blot showed that serum starvation pretreatment significantly reduced the expression levels of LC3II and p62 induced by H_2_O_2_ (Figure 2D). There was no remarkable difference in p62 levels between serum starvation pretreatment group and control group, suggesting that autophagy flux inhibition induced by H_2_O_2_ was restored. Furthermore, serum starvation pretreatment significantly prevented H_2_O_2_-induced reduction of cell viability (*p* < 0.001, Figure 2E). In addition, serum starvation pretreatment remarkably reduced H_2_O_2_-induced ROS generations and mitochondrial dysfunction (Figure S1). These results suggest that H_2_O_2_-induced oxidative damage was via autophagy dysregulation in PC12 cells.

### 3.3. Characterization of BSA-MnO_2_

**Figure 3:**
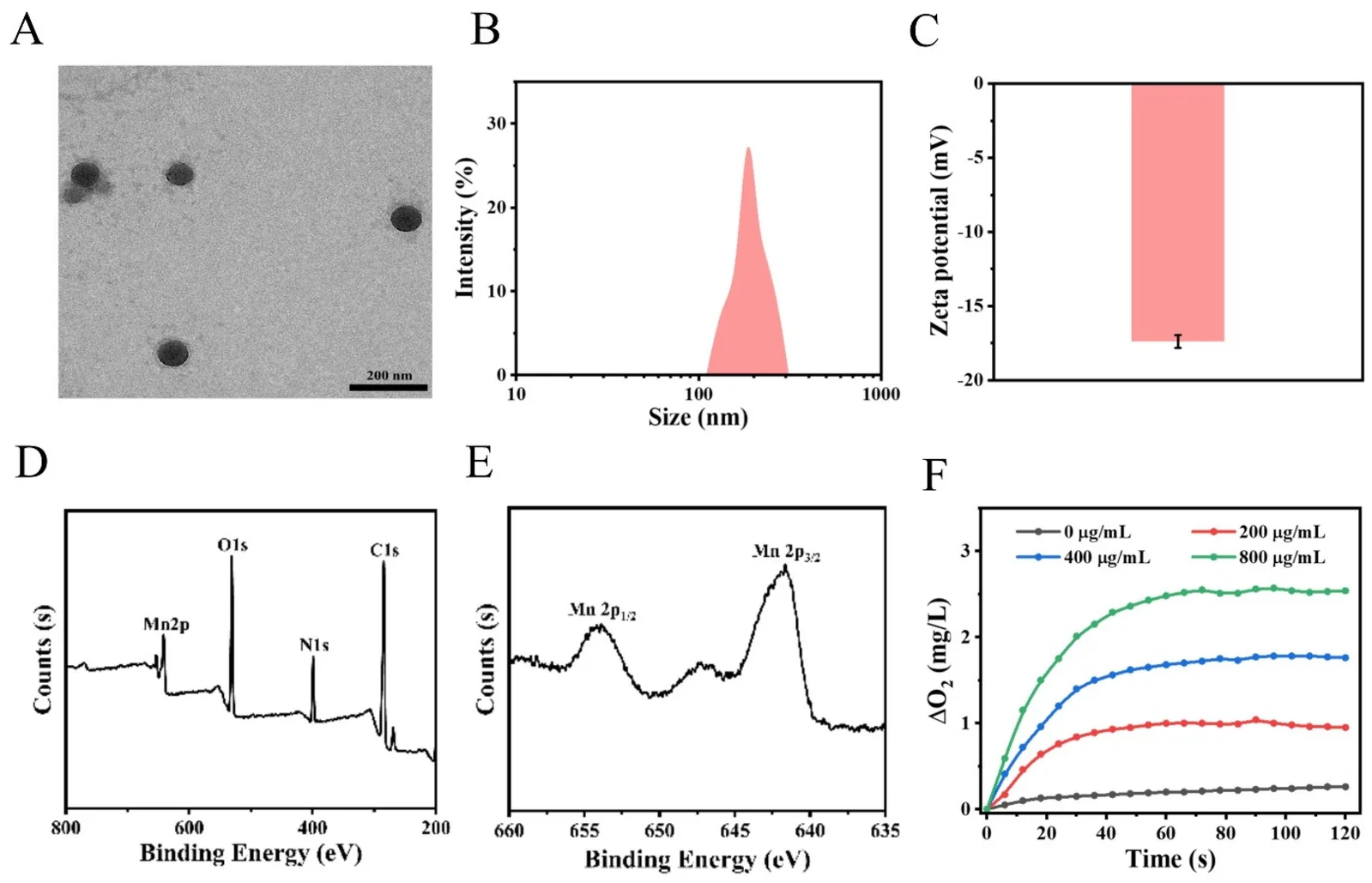
Characterizations of BSA-MnO_2_. (**A**) Representative TME images of BSA-MnO_2_. Scale bar = 100 nm. (**B**) Hydrodynamic size distribution and (**C**) Zeta potential of BSA-MnO_2_. (**D**) XPS spectrum of BSA-MnO_2_. (**E**) High-resolution Mn 2p XPS spectrum of BSA-MnO_2_. (**F**) The changes in O_2_ concentration in 100 × 10^−6^ M H_2_O_2_ after BSA-MnO_2_ in different concentrations was added, and the changes in the concentration of O_2_ generated by H_2_O_2_ were used as a control. Data are reported as mean ± SEM from three independent experiments.

BSA-MnO_2_ was prepared via an oxidation-reduction method [23]. In brief, BSA-MnO_2_ was prepared by directly mixing the solution of MnCl_2_ and BSA, which was adjusted by NaOH to pH 11.0. TEM images showed the spherical structure of prepared BSA-MnO_2_ (Figure 3A). The size of nanoparticles was calculated, and the hydrodynamic diameter of BSA-MnO_2_ was about 150 nm in a magnified image (Figure 3B). We next determined long-term colloidal stability of BSA-MnO_2_ via zeta potential. Our data showed that the zeta-potential of BSA-MnO_2_ was −17.38 ± 0.43 mV (Figure 3C), which indicated the colloidal stability of BSA-MnO_2_. Furthermore, XPS was applied to detect Mn, O, N and C elements, and the results showed that Mn 2p XPS spectrum of BSA-MnO_2_ was assigned to Mn 2p_1/2_ at 653.8 eV and Mn 2p_3/2_ at 642.2 eV, suggesting that the element Mn existed in the tetravalent state in the nanoparticles (Figure 3D,E). These results revealed that BSA was successfully encapsulated on the surface of MnO_2_ nanospheres. As MnO_2_ could catalyze H_2_O_2_ into O_2_, we investigated the catalytic activity of prepared BSA-MnO_2_ by measuring the amount of residual H_2_O_2_ after incubation with different concentrations of BSA-MnO_2_ for a specific time. As shown in Figure 3F, BSA-MnO_2_ could efficiently scavenge H_2_O_2_, and the elimination ability was concentration-dependent and time-dependent. Taken together, the above results indicate that the prepared BSA-MnO_2_ is able to induce potential therapeutic effects against oxidative stress damage.

### 3.4. BSA-MnO_2_ Prevents H_2_O_2_-Induced Oxidative Damage in PC12 Cells

**Figure 4:**
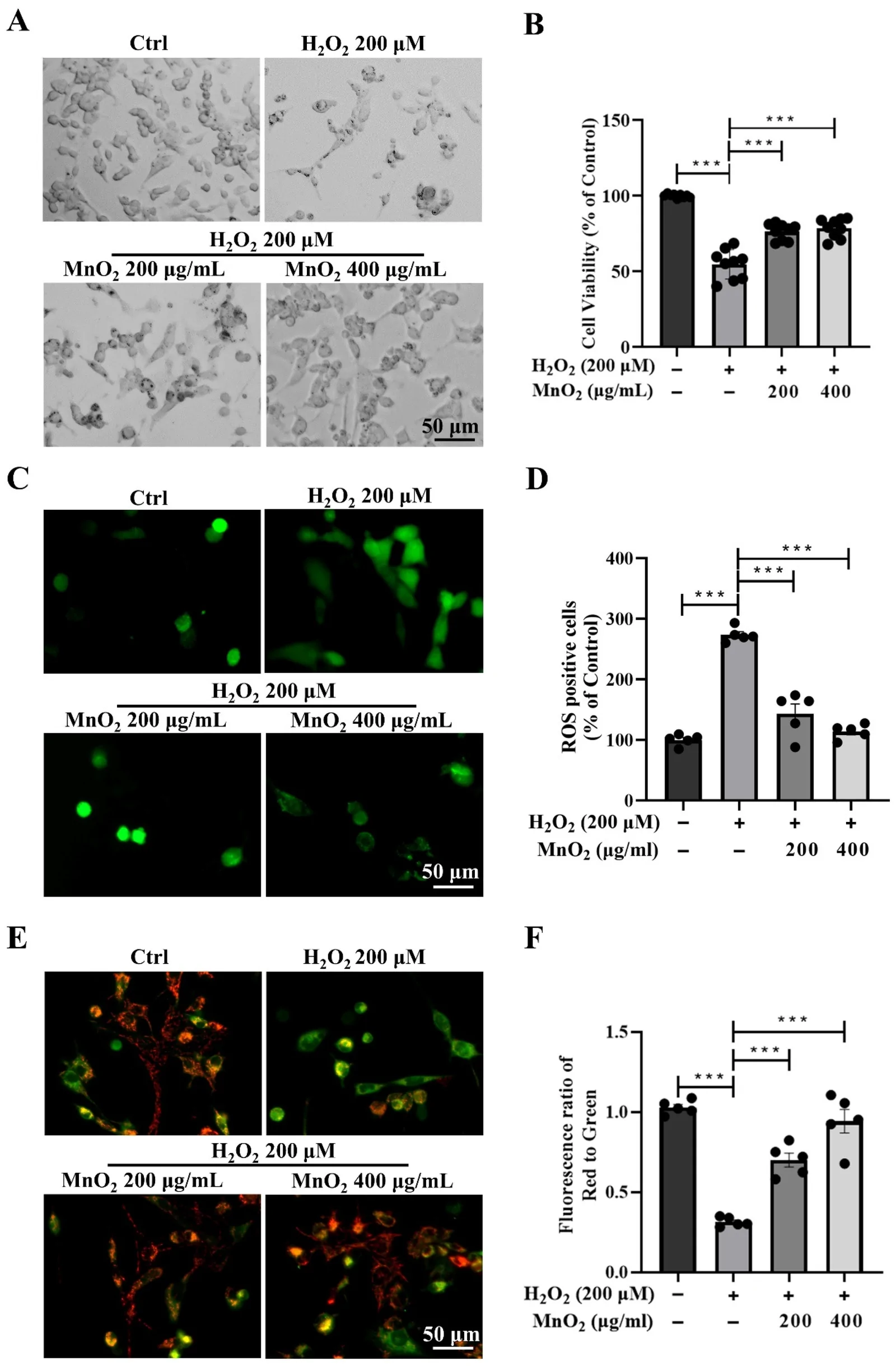
BSA-MnO_2_ prevents H_2_O_2_-induced oxidative damage in PC12 cells. Cultured PC12 cells were incubated in different concentrations (200 and 400 μg/mL) of BSA-MnO_2_ for 30 min, followed by the application of H_2_O_2_ (200 μM) for another 24 h. (**A**) Representative images of the cell morphology. Scale bar = 50 μm. (**B**) Cell viability was calculated using CCK-8 kit. (**C**) Treated PC12 cells were stained with DCFH-DA, and fluorescent density mean of ROS level was calibrated. 6-well plates with cells were observed under a fluorescent microscope. Scale bar = 50 μm. (**D**) Fluorescent density mean of ROS level was calibrated. (**E**) All treated PC12 cells were stained with JC-1 kit, and (**F**) ratios of JC-1 red/green fluorescence optical density was analyzed by ImageJ software. Scale bar = 50 μm. All data in bar charts represent mean ± SEM from at least three independent experiments. *** *p* <  0.001 versus the H_2_O_2_ group or the control group (Ctrl).

To further evaluate the biological effects of BSA-MnO_2_, H_2_O_2_-induced oxidative damage was employed in PC12 cells. PC12 cells were exposed to 200 μM H_2_O_2_ in the presence or absence of various concentrations (50, 100, 200, 400, and 800 μg/mL) of BSA-MnO_2_ for 24 h, respectively. As shown in Figure S2A, all concentrations of BSA-MnO_2_ significantly prevented H_2_O_2_-induced reduction of cell viability compared to the H_2_O_2_ group (*p* < 0.001), but the neuroprotective effect of BSA-MnO_2_ at 800 μg/mL showed a reduction compared to that at 400 μg/mL (*p* < 0.01). The reduction in neuroprotective effects of 800 μg/mL BSA-MnO_2_ suggested that the higher concentration of BSA-MnO_2_ could be toxic to PC12 cells. Therefore, 200 μg/mL and 400 μg/mL BSA-MnO_2_ were applied for the follow experiments. The morphological images showed that 200 and 400 μg/mL BSA-MnO_2_ significantly prevented cell loss induced by H_2_O_2_ and increased cell viability (Figure 4A,B). Furthermore, ROS production induced by 200 μM H_2_O_2_ was significantly prevented by both 200 μg/mL and 400 μg/mL BSA-MnO_2_ (*p* < 0.001, Figure 4C,D). Moreover, JC-1 staining showed that the decrease in the red/green ratio induced by 200 μM H_2_O_2_ was substantially reversed by the administration of BSA-MnO_2_ at 200 μg/mL and 400 μg/mL concentrations (Figure 4E, *p* < 0.001), which indicates that BSA-MnO_2_ prevented mitochondrial membrane potential disruption induced by H_2_O_2_. In addition, our patch-clamp recordings showed that the *I*_Na_ normalized by cell capacitance was dramatically reduced in H_2_O_2_-treated PC12 cells in its peak amplitude and in current density. The median value of current amplitude was −19.51 pA in the H_2_O_2_ group but −42.8 pA in the BSA-MnO_2_-treated group (Figure S2B). *I*-*V* curves further showed a reduced current density in the BSA-MnO_2_-treated group compared to the H_2_O_2_ group, but there was no difference between the control group and the treated group (Figure S2C). H_2_O_2_-induced cell membrane potential damage could be restored by BSA-MnO_2_ treatments. Taken together, the above results suggest that BSA-MnO_2_ prevents H_2_O_2_-induced oxidative damage, including inhibiting cell loss, decreasing ROS production, restoring mitochondrial functions and repaired cell membrane functions, in PC12 cells.

### 3.5. BSA-MnO_2_ Is Neuroprotective Against H_2_O_2_-Induced Oxidative Damage via Autophagy

**Figure 5:**
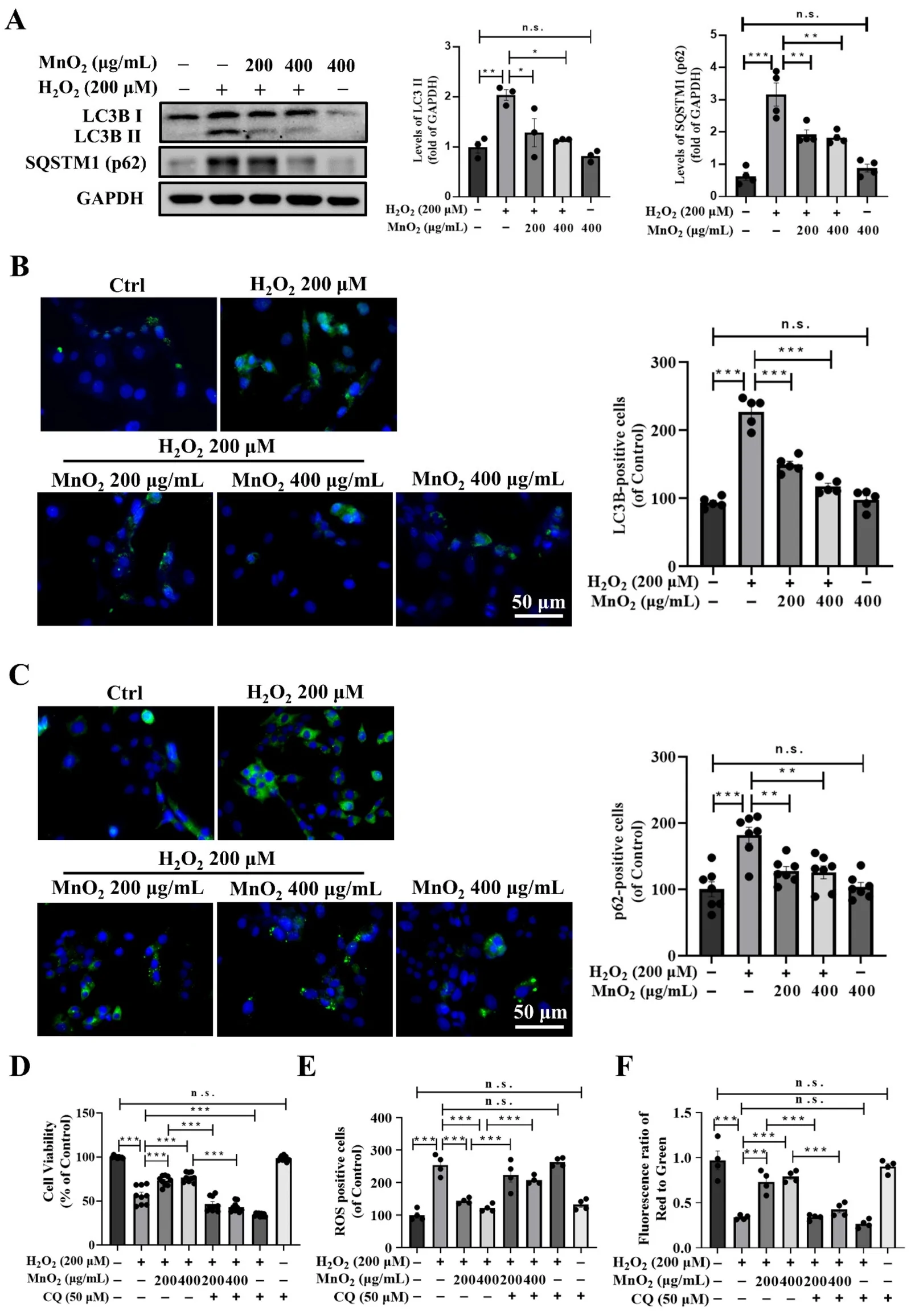
BSA-MnO_2_ protect against H_2_O_2_-induced oxidative damage via restoring autophagy. (**A**) The protein expression levels of LC3 and p62 were determined by Western blot in PC12 cells treated with or without BSA-MnO_2_ (200 and 400 μg/mL) for 30 min, then in the presence or absence of H_2_O_2_ (200 μM), and expression of GAPDH served as loading control. Quantitation of LC3 and p62 expression levels was analyzed. (**B**) LC3II detected using immunocytochemistry was photographed under fluorescent microscope, and fluorescent density of LC3II was calibrated. Scale bar = 50 μm. (**C**) p62 detected using immunocytochemistry was photographed under fluorescent microscope, and fluorescent density of p62 was calibrated. Scale bar = 50 μm. (**D**) Cell viability was detected. (**E**) The fluorescent density mean of the ROS level was calibrated. (**F**) The ratios of JC-1 red/green fluorescence optical density were analyzed by ImageJ software. All data in bar charts represent mean ± SEM from at least three independent experiments. * *p* <  0.05, ** *p* <  0.01, *** *p* <  0.001 versus the H_2_O_2_ group or the control group (Ctrl). n.s., no significance.

To investigate whether the neuroprotection of BSA-MnO_2_ against H_2_O_2_ was related to autophagy in PC12 cells, we analyzed the protein levels of LC3II and p62 [24]. Our results showed that exposure to H_2_O_2_ remarkably increased the protein levels of LC3II and p62 compared to the control group (*p* < 0.001), whereas the treatment with BSA-MnO_2_ could remarkably reduce the protein levels of LC3II and p62 (Figure 5A). Similarly, the immunofluorescence results showed BSA-MnO_2_ treatments decreased LC3II and p62 expression induced by H_2_O_2_ (Figure 5B,C). These data indicate BSA-MnO_2_ restored autophagy functions. As shown in Figure 5D, a cell viability assay further demonstrated that the neuroprotective effect of BSA-MnO_2_ against H_2_O_2_ was remarkably prevented by an inhibitor of autophagy, CQ. Also, the reduced ROS generation and recovered mitochondrial membrane potential induced by BSA-MnO_2_ against H_2_O_2_ were abolished by CQ (Figure 5E,F and Figure S3). Taken together, these results suggest that BSA-MnO_2_ prevents H_2_O_2_-induced oxidative stress damage via restoring autophagy functions.

### 3.6. PI3K/Akt Signaling Pathway Is Involved in the Neuroprotective Effects of BSA-MnO_2_

**Figure 6:**
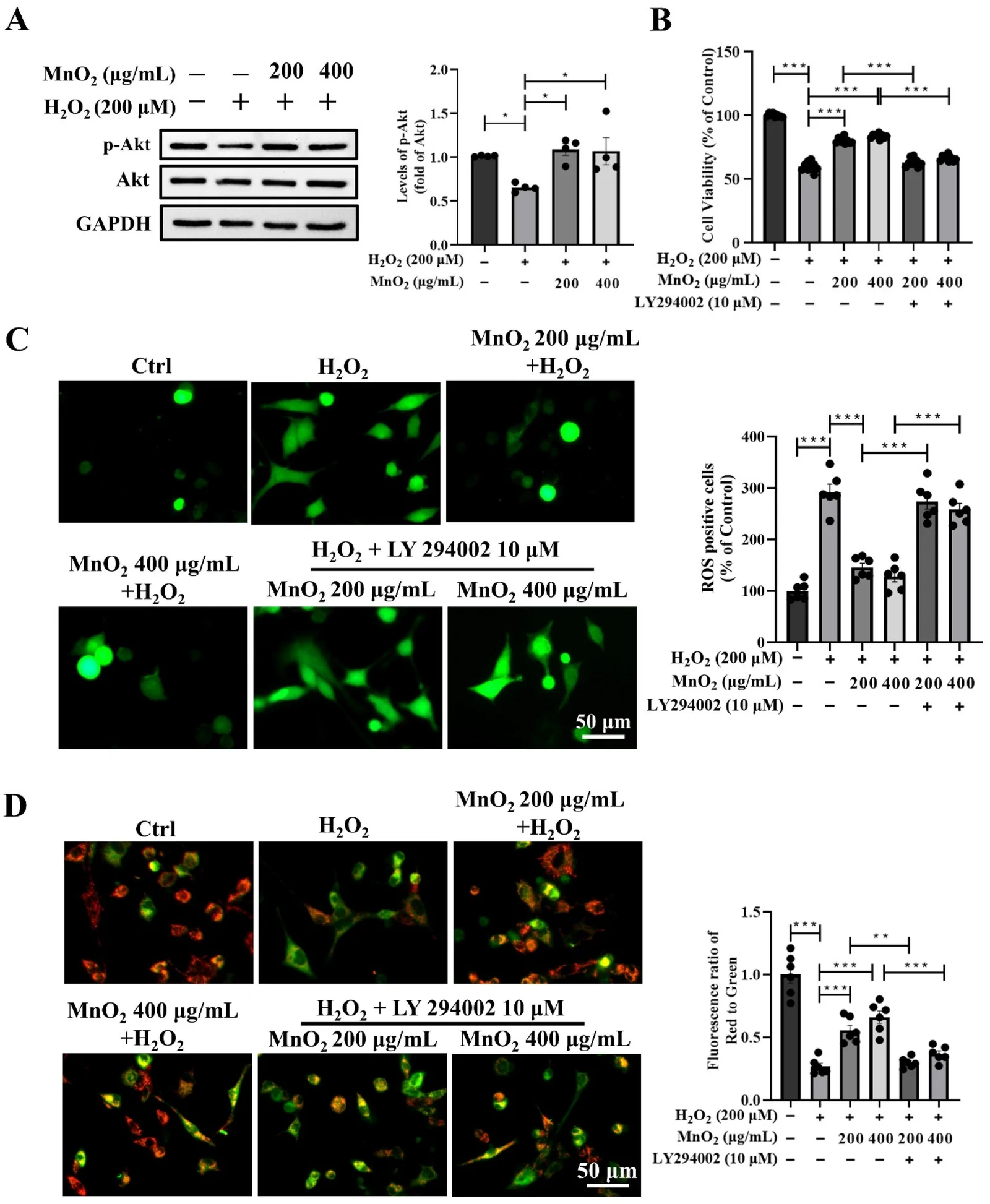
PI3K/Akt signaling pathway is involved in the neuroprotective effects of BSA-MnO_2_. (**A**) The expression levels of p-Akt and Akt were determined, and expression of GAPDH served as loading control. Quantitation of p-Akt expression level was analyzed. (**B**) Cell viability was detected. (**C**) Treated PC12 cells were stained with DCFH-DA, and fluorescent density mean of ROS level was calibrated. Scale bar = 50 μm. (**D**) Treated PC12 cells were stained with JC-1 kit, and the ratios of JC-1 red/green fluorescence optical density were analyzed by ImageJ software. Scale bar = 50 μm. All data in bar charts represent mean ± SEM from at least three independent experiments. * *p* <  0.05, ** *p* <  0.01, *** *p* <  0.001 versus the H_2_O_2_ group or the control group (Ctrl).

The PI3K/Akt signaling pathway has been implicated in oxidative damage-mediated neurodegeneration [25]. Herein, we investigated the potential role of the PI3K/Akt signaling pathway in the protective response of BSA-MnO_2_ against H_2_O_2_-induced oxidative stress damage. The protein levels of phosphorylated Akt (p-Akt) were monitored. We found that the stimulation of H_2_O_2_ significantly downregulated the level of p-Akt, whereas the p-Akt level could be restored by the treatment with BSA-MnO_2_ (Figure 6A), indicating that the PI3K/Akt signaling pathway was involved in the neuroprotective effects of BSA-MnO_2_ against H_2_O_2_. Furthermore, the CCK-8 assay showed that the increase in cell viability mediated by BSA-MnO_2_ was almost abolished by LY294002, a selective PI3K inhibitor (Figure 6B). As shown in Figure 6C, LY294002 significantly increased ROS generation in PC12 cells treated with BSA-MnO_2_ against H_2_O_2_. In addition, LY294002 inhibited the recovery of mitochondrial membrane potential mediated by BSA-MnO_2_ (Figure 6D). LY294002 at 10 μM significantly decreased the levels of p-Akt upregulated by BSA-MnO_2_ (Figure S4). Taken together, these results suggest that the activated PI3K/Akt signaling is involved in the neuroprotective effects of BSA-MnO_2_ against H_2_O_2_-induced oxidative damage.

### 3.7. BSA-MnO_2_ Regulates Autophagy-Mediated Neuroprotection via PI3K/Akt Signaling Pathway

**Figure 7:**
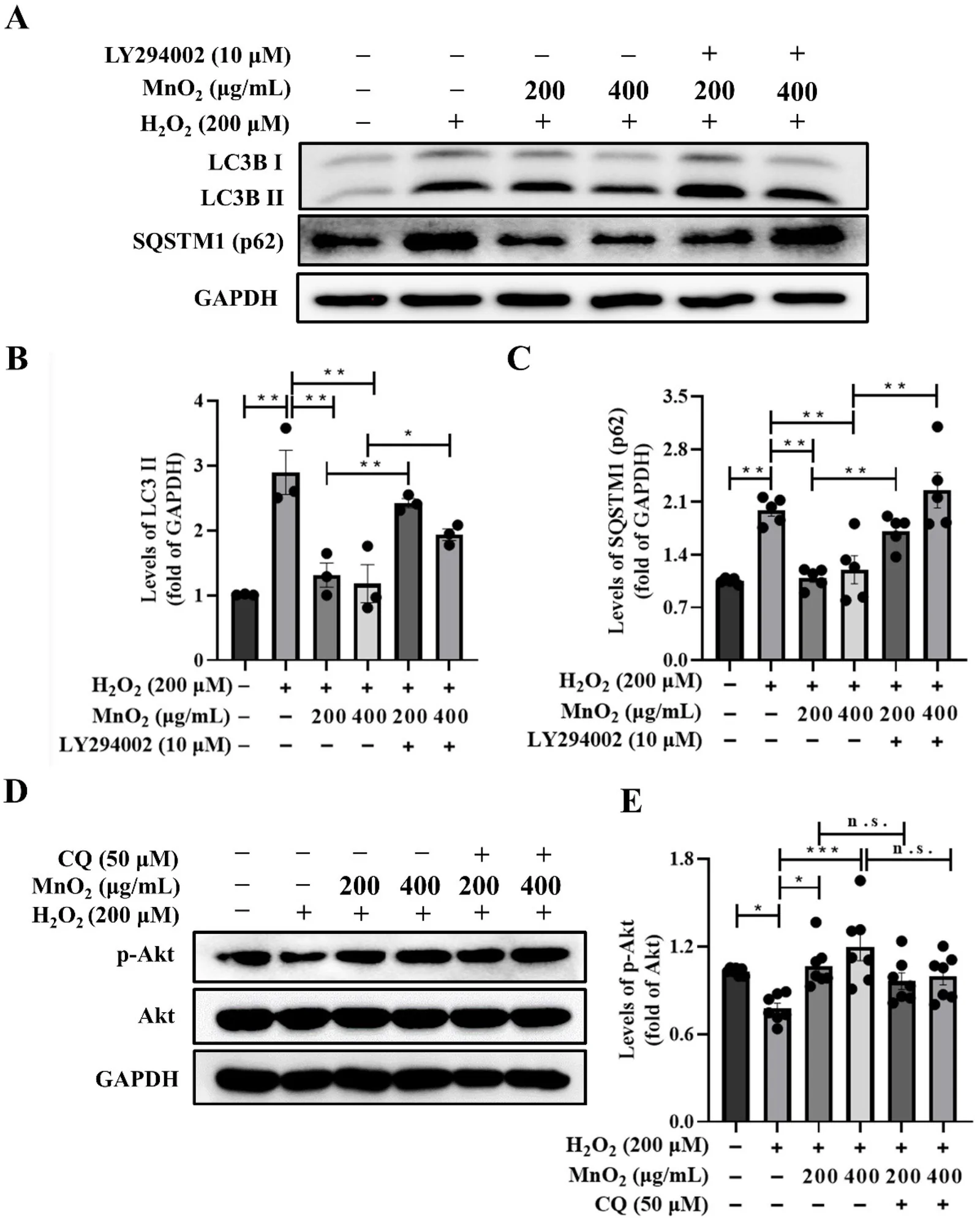
BSA-MnO_2_ protects PC12 cells against H_2_O_2_-induced oxidative damage via PI3K/Akt signaling pathway to restore autophagy. PC12 cells were treated with or without BSA-MnO_2_ (200 and 400 μg/mL) for 30 min, then in the presence or absence of LY294002 (10 μM) or in the presence or absence of CQ for 30 min, followed by H_2_O_2_ (200 μM). The control group consisted of the untreated cells. (**A**) The protein expression levels of LC3 and p62 were determined by Western blot, and the quantitation of (**B**) LC3II and (**C**) p62 was calibrated. Expression of GAPDH served as loading control. (**D**) The protein expression levels of p-Akt and Akt were determined by Western blot. Expression of GAPDH served as loading control. (**E**) Quantitation of p-Akt expression was calibrated. All data in bar charts represent mean ± SEM from at least three independent experiments. * *p* <  0.05, ** *p* <  0.01, *** *p* <  0.001 versus the H_2_O_2_ group or the control group (Ctrl). n.s., no significance.

In order to clarify the neuroprotective mechanism of BSA-MnO_2_ against H_2_O_2_, further research was conducted on the relation between autophagy and PI3k/Akt signaling pathway. We analyzed the protein levels of LC3II and p62 in PC12 cells treated with H_2_O_2_ and BSA-MnO_2_ in the presence or absence of LY294002. The results showed that compared with the BSA-MnO_2_ treatment group, the application of LY294002 almost completely prevented the effects of BSA-MnO_2_ that reversed the levels of LC3II and p62 (Figure 7A–C). However, CQ did not affect the expression levels of p-Akt mediated by BSA-MnO_2_ (Figure 7D,E). Accordingly, we conclude that BSA-MnO_2_ activates the PI3K/Akt signaling pathway to restore autophagy functions against H_2_O_2_-mediated oxidative damage.

## 4. Discussion

Accumulating studies have suggested the neuroprotective effects of MnO_2_ nanoparticles in the central nervous system (CNS). A previous study showed that a MnO_2_ nanoparticle-dotted hydrogel promoted spinal cord repair by improving the ROS microenvironment [18]. Macrophage-disguised FIY-loaded MnO_2_ nanoparticles have also been shown to efficiently consume excessive ROS and attenuate proinflammation from ischemic stroke [21]. In this study, we demonstrated that BSA-MnO_2_ NPs restored autophagy though the PI3K/Akt signaling pathway, thereby protecting PC12 cells from H_2_O_2_-induced oxidative damage. Collectively, these findings indicate that MnO_2_ nanoparticles could serve as a novel therapeutic strategy for neurodegeneration.

Oxidative stress is a pivotal driver of cellular damage in a wide spectrum of acute and chronic neurodegenerative disorders [26]. The cellular damage caused by oxidative stress is mainly due to the excessive production of ROS, which triggers DNA damage, mitochondrial dysfunction, and protein degeneration in neuronal cells [27,28]. H_2_O_2_ is commonly used as an in vitro inducer of oxidative stress to study the neuroprotective effects of bioactive molecules against oxidative damage in neuronal cells. Evidence showed that 250 μM H_2_O_2_ could significantly disrupt cell morphology and stimulate excessive ROS production [29], which was consistent with our experimental observations. Furthermore, H_2_O_2_ exposure induced mitochondrial damage. Excessive ROS accumulation upon H_2_O_2_ stimulation led to the loss of mitochondrial membrane potential and ultimately initiated mitochondria-dependent neuronal cell death [30]. Our experimental results verified that H_2_O_2_ significantly reduced mitochondrial membrane potential (Figure 1E,F), which is consistent with previous studies [31,32]. Moreover, H_2_O_2_-induced oxidative stress could modulate the activity of multiple ion channels in neuronal cells, including activating native L-type Ca^2+^ channels [33] and Cl^−^ channels [34], promoting intracellular Ca^2+^ current influx [34] and Cl^−^ current efflux [35]. Additionally, H_2_O_2_ directly affects Na^+^ current via altering the membrane potential and transmembrane ion flux [36]. Consistent with these findings, our patch clamp electrophysiological recordings revealed that H_2_O_2_ significantly reduced Na^+^ current compared with the control group (Figure S2B,C). Taken together, our results demonstrated that H_2_O_2_ exposure caused mitochondrial membrane depolarization, increased ROS levels, and decreased cell viability in PC12 cells.

Autophagy is crucial for neuronal homeostasis and synaptic plasticity under normal physiological conditions [37,38]. Mounting evidence suggests that activated autophagy could not only prevent the progress of AD by promoting the metabolism of Aβ [39,40], but also improve PD through some associated genes [41], indicating that autophagy maintains neuronal survival. Notably, autophagy impairment contributes to the initiation and exacerbation of both PD and AD processes [42,43], and oxidative stress is recognized as a key regulator of autophagy impairment and mitochondrial dysfunction [44]. These studies suggest that selective agonists of autophagy could serve as a potential strategy for the treatment of neurodegenerative disorders. Our study showed that H_2_O_2_ dose-dependently increased the levels of both LC3II and p62 in PC12 cells (Figure 2), which was supported by previous studies [24,45]. These experimental results indicated that H_2_O_2_ might induce autophagic defects via blocking the fusion of autophagosomes and lysosomes. Serum starvation pretreatment effectively reversed H_2_O_2_-induced cell viability loss, reduced ROS overproduction, and restored mitochondrial membrane potential in PC12 cells, further verifying the involvement of autophagic defects in H_2_O_2_-mediated neuronal oxidative damage. Consistent with our results, Tai et al. [44] reported that H_2_O_2_-induced autophagy impairment hindered fusion with lysosomal and mitochondrial dysfunction in NIH3T3 cells. Another study reported that autophagy mediated H_2_O_2_-induced oxidative stress, including accumulated ROS generation and mitochondrial dysfunction in PC12 cells [46], which is consistent with our study. On the contrary, autophagy activation could also exacerbate neuronal damage under certain conditions. For instance, autophagy activated by PINK1 and Parkin is a potential pathological mechanism of PD [47]. The above studies suggest that autophagy activation could mediate opposite effects under different conditions. Our research showed that autophagy injury was involved in H_2_O_2_-induced oxidative damage in PC12 cells.

MnO_2_ is able to consume ROS and ameliorate the proinflammatory microenvironment. For instance, polyethylene glycol-modified MnO_2_ (PEG-MnO_2_) NPs have been demonstrated to protect cartilage against oxidative stress-induced inflammation [15]. Additionally, a MnO_2_ nanoparticle-dotted hydrogel alleviated ROS generation and repaired spinal cord injury [16]. Moreover, macrophage-disguised FIY-loaded MnO_2_ nanoparticles could efficiently consume excessive ROS, thereby exerting neuroprotective effects against ischemic stroke [17]. In the present study, BSA-templated manganese dioxide nanoparticles (BSA-MnO_2_ NPs) were fabricated by directly mixing MnCl_2_ with BSA as previous described [48]. For in vitro validation, PC12 cells were incubated in BSA-MnO_2_ NPs for 24 h and rinsed with PBS, followed by exposure to 200-μM H_2_O_2_ for another 24 h. Our experimental results showed that the pretreatment with BSA-MnO_2_ NPs effectively prevented a reduction in cell viability, an increase in ROS production, and a reduction in mitochondrial membrane potential induced by H_2_O_2_ in PC12 cells (Figure 4). These findings are consistent with a previous study reporting the antioxidant capacity of BSA-MnO_2_ against oxidative damage in BEAS-2B cells [49]. Notably, we further found that the protective effects of BSA-MnO_2_ NPs were mediated by autophagy (Figure 5). Patch-clamp electrophysiological assays further verified that the pretreatment with BSA-MnO_2_ recovered the impaired Na^+^ current in H_2_O_2_-treated PC12 cells (Figure S2B,C), which is in line with a previous study on the regulatory effects of BSA-MnO_2_ on cellular Na^+^ current [50]. CQ, a classic autophagy inhibitor, significantly suppressed the neuroprotective effects of BSA-MnO_2_ against oxidative damage in PC12 cells (Figure 5D,E and Figure S3). In particular, CQ accelerated H_2_O_2_-induced oxidative damage. Together, these results indicate that BSA-MnO_2_ NPs could attenuate H_2_O_2_-induced oxidative damage in PC12 cells by restoring functional autophagy. Further investigations are warranted to elucidate the precise autophagy-related signaling mechanisms underlying this protective effect.

However, evidence has shown that MnO_2_ NPs degrade under acidic microenvironments and high-concentration H_2_O_2_, thereby releasing free Mn^2+^ [51]. Excessive manganese ions tend to accumulate in brain tissue and trigger neuronal apoptosis, and long-term Mn accumulation causes neurological impairment [52]. Consistent with these findings, our results verified the neuroprotective effects of BSA-MnO_2_ NPs in H_2_O_2_-injured PC12 cells; nevertheless, a high concentration of 800 μg/mL BSA-MnO_2_ NPs markedly reduced the cell viability of PC12 cells (Figure S2A). These results indicate that BSA-MnO_2_ NPs exerted dual roles. Over-dosage of BSA-MnO_2_ NPs could trigger overactivated autophagy and subsequent autophagic cell death. Meanwhile, high-dose BSA-MnO_2_ NPs might disturb the homeostasis of the autophagy-associated signaling pathways, such as the AMPK or ERK1/2-MAPK pathway, disrupt the intrinsic balance between cell survival and apoptosis, and thus produce bidirectional adverse effects. Accordingly, further modification of BSA-MnO_2_ NPs is required to mitigate their toxic side-effects.

The PI3K/Akt signaling pathway has been well documented to activate autophagy [53,54]. In the current study, we further validated that the neuroprotection of BSA-MnO_2_ against H_2_O_2_-induced oxidative damage was closely related to the PI3K/Akt signaling pathway in PC12 cells (Figure 6 and Figure 7). BSA-MnO_2_ could recover the expression levels of p-Akt down-regulated by H_2_O_2_, and the neuroprotective effects of BSA-MnO_2_ could be completely abrogated by LY294002, a specific PI3K antagonist (Figure 6). Moreover, LY294002 increased the expression levels of both LC3II and p62 down-regulated by BSA-MnO_2_. In contrast, CQ could not alter the expression levels of p-Akt (Figure 7). Collectively, these results suggest that activation of the PI3k/Akt signaling pathway regulated autophagy and contributed to the neuroprotection provided by BSA-MnO_2_ against oxidative damage in PC12 cells. Consistently, activation of the PI3K/Akt signaling pathway was involved in protecting against H_2_O_2_-induced neurotoxicity in naked mole-rat skin fibroblasts [55,56]. Similarly, the PI3K/Akt/mTOR signaling pathway could attenuate H_2_O_2_-induced oxidative damage in HTR8 cells [57]. In a H_2_O_2_-induced oxidative-stress model of PC12 cells, we confirmed that BSA-MnO_2_ NPs exert neuroprotection by modulating autophagy via the PI3K cascade. Nevertheless, autophagy modulation is a complex biological process involving multiple signaling pathways. Multiple signaling pathways, including JNK, AMPK, Erk1/2, MAPK, as well as p38, have been reported to be involved in regulating the balance of autophagy and apoptosis [58]. For instance, AMPK suppresses the activity of mTORC1 to trigger ULK1-dependent autophagy, and the MAPK or Erk1/2 signaling pathway also participates in autophagic regulation. In the present study, these pathways were not explored in depth. Further investigations are required to clarify whether several signaling cascades regulate autophagy independently or in coordination.

Certainly, the current study investigated the neuroprotective effects of BSA-MnO_2_ NPs in PC12 cells using an acute oxidative stress model induced by H_2_O_2_, which fails to fully mimic the complex pathophysiological conditions of neurodegenerative diseases. The potential role of BSA-MnO_2_ NPs as a candidate against oxidative damage needs to be further explored through a combination of in vitro and in vivo experiments in the future. For instance, we will further explore the neuroprotective effects, underlying mechanisms and pharmacokinetic profiles of BSA-MnO_2_ NPs via using primary neurons and animal models. Moreover, we will systematically investigate manganese ion release, intracellular accumulation, long-term toxicity, in vivo biodistribution, and blood–brain barrier penetration ability, as well as drug metabolism and excretion pathways.

In summary, we clearly demonstrated that BSA-MnO_2_ protects PC12 cells from H_2_O_2_-induced oxidative damage by activating the PI3K/Akt signaling pathway to restore autophagy. Our research findings could provide new insights into the neuroprotective effects of BSA-MnO_2_.

## Data Availability

The original contributions presented in this study are included in the article/Supplementary Material. Further inquiries can be directed to the corresponding author.

## Supplementary Materials

The following supporting information can be downloaded at: https://www.mdpi.com/article/10.3390/pharmaceutics18091156/s1, Figure S1. Serum starvation reduces ROS production and mitochondrial dysfunction. Figure S2. BSA-MnO_2_ prevents H_2_O_2_-induced oxidative damage in PC12 cells. Figure S3. The recovered mitochondrial membrane potential and reduced ROS generations mediated by BSA-MnO_2_ against H_2_O_2_ were abolished by CQ. Figure S4. Expression of p-Akt up-regulated by BSA-MnO_2_ is decreased by Ly294002. Figure S5. The schematic diagram of the neuroprotection of BSA-MnO_2_ against H_2_O_2_-induced oxidative damage in PC12 cells.

## References

[1] Sanford S.L., Welfer G.A., Freudenthal B.D., Opresko P.L. (2020). Mechanisms of telomerase inhibition by oxidized and therapeutic dNTPs. Nat. Commun..

[2] Riaz Z., Richardson G.S., Jin H., Zenitsky G., Anantharam V., Kanthasamy A., Kanthasamy A.G. (2024). Nuclear pore and nucleocytoplasmic transport impairment in oxidative stress-induced neurodegeneration: Relevance to molecular mechanisms in Pathogenesis of Parkinson’s and other related neurodegenerative diseases. Mol. Neurodegener..

[3] Bai R., Guo J., Ye X.Y., Xie Y., Xie T. (2022). Oxidative stress: The core pathogenesis and mechanism of Alzheimer’s disease. Ageing Res. Rev..

[4] Lei L., Zou Z., Liu J., Xu Z., Fu Y., Tian Y., Zhang W. (2021). Multifunctional peptide-assembled micelles for simultaneously reducing amyloid-beta and reactive oxygen species. Chem. Sci..

[5] Gahtani R.M., Shoaib S., Hani U., Jayachithra R., Alomary M.N., Chauhan W., Jahan R., Tufail S., Ansari M.A. (2024). Combating Parkinson’s disease with plant-derived polyphenols: Targeting oxidative stress and neuroinflammation. Neurochem. Int..

[6] Lee Y.M., He W., Liou Y.C. (2021). The redox language in neurodegenerative diseases: Oxidative post-translational modifications by hydrogen peroxide. Cell Death Dis..

[7] Liu L., Zhang K., Sandoval H., Yamamoto S., Jaiswal M., Sanz E., Li Z., Hui J., Graham B.H., Quintana A. (2015). Glial lipid droplets and ROS induced by mitochondrial defects promote neurodegeneration. Cell.

[8] Li R.L., Zhang Q., Liu J., Sun J.-Y., He L.-Y., Duan H.-X., Peng W., Wu C.-J. (2020). Hydroxy-alpha-sanshool possesses protective potentials on H_2_O_2_-stimulated PC12 cells by suppression of oxidative stress-induced apoptosis through regulation of PI3K/Akt signal pathway. Oxid. Med. Cell Longev..

[9] Wan T., Wang Z., Luo Y., Zhang Y., He W., Mei Y., Xue J., Li M., Pan H., Li W. (2019). FA-97, a new synthetic caffeic acid phenethyl ester derivative, protects against oxidative stress-mediated neuronal cell apoptosis and scopolamine-induced cognitive impairment by activating Nrf2/HO-1 signaling. Oxid. Med. Cell. Longev..

[10] Yang J., Fang L., Lu H., Liu C., Wang J., Wu D., Min W. (2022). Walnut-derived peptide enhances mitophagy via JNK-mediated PINK1 activation to reduce oxidative stress in HT-22 cells. J. Agric. Food Chem..

[11] Ji S.T., Kim Y.J., Jung S.Y., Kim D.Y., Kang S., Park J.H., Jang W.B., Ha J., Yun J., Kwon S.-M. (2018). Oleuropein attenuates hydrogen peroxide-induced autophagic cell death in human adipose-derived stem cells. Res. Commun..

[12] Zhang X., Bao M., Zhang J., Zhu L., Wang D., Liu X., Xu L., Luan L., Liu Y., Liu Y. (2024). Neuroprotective mechanism of ribisin A on H_2_O_2_-induced PC12 cell injury model. Tissue Cell.

[13] Xiong Y., Zhou D., Zheng K., Bi W., Dong Y. (2022). Extracellular ATP binding to P2Y1 receptors prevents glutamate-induced excitotoxicity: Involvement of Erk1/2 signaling pathway to suppress autophagy. Front. Neurosci..

[14] Prasad P., Gordijo C.R., Abbasi A.Z., Maeda A., Ip A., Rauth A.M., DaCosta R.S., Wu X.Y. (2014). Multifunctional albumin-MnO_2_ nanoparticles modulate solid tumor microenvironment by attenuating hypoxia, acidosis, vascular endothelial growth factor and enhance radiation response. ACS Nano.

[15] Kumar S., Adjei I.M., Brown S.B., Liseth O., Sharma B. (2019). Manganese dioxide nanoparticles protect cartilage from inflammation-induced oxidative stress. Biomaterials.

[16] Li L., Xiao B., Mu J., Zhang Y., Zhang C., Cao H., Chen R., Patra H.K., Yang B., Feng S. (2019). A MnO_2_ nanoparticle-dotted hydrogel promotes spinal cord repair via regulating reactive oxygen species microenvironment and synergizing with mesenchymal stem cells. ACS Nano.

[17] Li C., Zhao Z., Luo Y., Ning T., Liu P., Chen Q., Chu Y., Guo Q., Zhang Y., Zhou W. (2021). Macrophage-disguised manganese dioxide nanoparticles for neuroprotection by reducing oxidative stress and modulating inflammatory microenvironment in acute ischemic stroke. Adv. Sci..

[18] Aschner M., Skalny A.V., Notova S.V., Tinkova M.N., Lu R., Skalny A.A., Santamaria A., Zhou J.-C., Tinkov A.A. (2026). Molecular mechanisms of manganese oxide nanoparticles toxicity in brain and other tissues: An overview. Front. Biosci..

[19] Akbar M.U., Guo W., Shen X., Fang Y., Liu Y., Wang P., Elnathan R., Chen Y. (2025). Rewiring neuroimmunity: Nanoplatform innovations for CNS disease therapy. Adv. Ther..

[20] Mu Z., Chen Y., Hu Y., Wang H., Li J. (2025). Tailored brain metastatic tumor cells-derived apoptotic bodies ameliorate Alzheimer’s disease by promoting microglia efferocytosis and neuroinflammation mitigation. J. Nanobiotechnol..

[21] Wang Y.T., Liu T.Y., Shen C.-H., Lin S.-Y., Hung C.-C., Hsu L.-C., Chen G.-C. (2022). K48/K63-linked polyubiquitination of ATG9A by TRAF6 E3 ligase regulates oxidative stress-induced autophagy. Cell Rep..

[22] Gao X., Xiong Y., Ma H., Zhou H., Liu W., Sun Q. (2025). Visualizing bulk autophagy in vivo by tagging endogenous LC3B. Autophagy.

[23] Du B., Ding X., Wang H., Du Q., Xu T., Huang J., Zhou J., Cheng G. (2019). Development of an interactive tumor vascular suppression strategy to inhibit multidrug resistance and metastasis with pH/H_2_O_2_ responsive and oxygen-producing nanohybrids. J. Mater. Chem. B.

[24] Huang X., Yao J., Liu L., Chen J., Mei L., Huangfu J., Luo D., Wang X., Lin C., Chen X. (2023). S-acylation of p62 promotes p62 droplet recruitment into autophagosomes in mammalian autophagy. Mol. Cell.

[25] Dash U.C., Bhol N.K., Swain S.K., Samal R.R., Nayak P.K., Raina V., Panda S.K., Kerry R.G., Duttaroy A.K., Jena A.B. (2025). Oxidative stress and inflammation in the pathogenesis of neurological disorders: Mechanisms and implications. Acta Pharm. Sin. B.

[26] Wen P., Sun Z., Gou F., Wang J., Fan Q., Zhao D., Yang L. (2025). Oxidative stress and mitochondrial impairment: Key drivers in neurodegenerative disorders. Ageing Res. Rev..

[27] Rehman M.U., Sehar N., Dar N.J., Khan A., Arafah A., Rashid S., Rashid S.M., Ganaie M.A. (2023). Mitochondrial dysfunctions, oxidative stress and neuroinflammation as therapeutic targets for neurodegenerative diseases: An update on current advances and impediments. Neurosci. Biobehav. Rev..

[28] Bhat A.H., Dar K.B., Anees S., Zargar M.A., Masood A., Sofi M.A., Ganie S.A. (2015). Oxidative stress, mitochondrial dysfunction and neurodegenerative diseases; a mechanistic insight. Biomed. Pharmacother..

[29] Chen X.H., Zhou X., Yang X.Y., Zhou Z.-B., Lu D.-H., Tang Y., Ling Z.-M., Zhou L.-H., Feng X. (2016). Propofol protects against H_2_O_2_-induced oxidative injury in differentiated PC12 cells via inhibition of Ca^2+^-dependent NADPH oxidase. Cell Mol. Neurobiol..

[30] Fu S.C., Liu J.M., Lee K.I., Tang F.-C., Fang K.-M., Yang C.-Y., Su C.-C., Chen H.-H., Hsu R.-J., Chen Y.-W. (2020). Cr(VI) induces ROS-mediated mitochondrial-dependent apoptosis in neuronal cells via the activation of Akt/ERK/AMPK signaling pathway. Toxicol. In Vitro.

[31] Mengual R., Bobo-Jiménez V., Rodríguez C., Lapresa R., García-Rodríguez D., Jiménez-Blasco D., Cabiscol E., Ros J., Delgado-Esteban M., Bolaños J.P. (2025). Endogenous mitochondrial hydrogen peroxide regulates neurogenesis during cortical development. Redox. Biol..

[32] Petricca S., Matrone A., Capece D., Flati I., Flati V., Ricevuto E., Celenza G., Franceschini N., Mastrangelo M., Pellegrini C. (2025). Pyrroloquinoline quinone (PQQ) attenuates hydrogen peroxide-induced injury through the enhancement of mitochondrial function in human trabecular meshwork cells. Int. J. Mol. Sci..

[33] Toda T., Yamamoto S., Yonezawa R., Mori Y., Shimizu S. (2016). Inhibitory effects of tyrphostin AG-related compounds on oxidative stress-sensitive transient receptor potential channel activation. Eur. J. Pharmacol..

[34] Wu F., Chi Y., Jiang Z., Xu Y., Xie L., Huang F., Wan D., Ni J., Yuan F., Wu X. (2020). Hydrogen peroxide sensor HPCA1 is an LRR receptor kinase in Arabidopsis. Nature.

[35] Muñoz-Palma E., Acosta-Tapia N., Villablanca C., Wilson C., Hidalgo C., González-Billault C. (2026). NMDAR and glutamate control axon growth by regulating Rac1-dependent actin dynamics and H_2_O_2_ production. Redox. Biol..

[36] Li J., Li Y., Liu Y., Yu H., Xu N., Huang D., Xue Y., Li S., Chen H., Liu J. (2021). Fibroblast growth factor 21 ameliorates Na_V_1.5 and Kir2.1 channel dysregulation in human AC16 cardiomyocytes. Front. Pharmacol..

[37] Kulkarni A., Chen J., Maday S. (2018). Neuronal autophagy and intercellular regulation of homeostasis in the brain. Curr. Opin. Neurobiol..

[38] Stavoe A.K.H., Holzbaur E.L.F. (2019). Autophagy in Neurons. Annu. Rev. Cell Dev. Biol..

[39] Zhu X.C., Yu J.T., Jiang T., Tan L. (2013). Autophagy modulation for Alzheimer’s disease therapy. Mol. Neurobiol..

[40] Li Q., Liu Y., Sun M. (2017). Autophagy and Alzheimer’s disease. Cell Mol. Neurobiol..

[41] Lu J., Wu M., Yue Z. (2020). Autophagy and Parkinson’s disease. Adv. Exp. Med. Biol..

[42] Pan J.-P., Wang P.-J., Zhang J., Knapskog A.-B., Watne L.O., Wang H.-L., Lagartos-Donate M.J., Lautrup S., Mao L.-P., Wang Q. (2026). Reduced ULK1 links impaired autophagy and mitophagy to Alzheimer’s disease pathology. Nat. Aging.

[43] Zhang Y., Jiang Y., Yu Z., Li Y., Zhang Z., Zheng F., Hu H., Yu G., Guo Z., Wu S. (2025). Characterizing microglial heterogeneity in autophagy impairment of Paraquat-induced Parkinson’s disease-like neurodegeneration. Ecotoxicol. Environ. Saf..

[44] Tai H., Wang Z., Gong H., Han X., Zhou J., Wang X., Wei X., Ding Y., Huang N., Qin J. (2017). Autophagy impairment with lysosomal and mitochondrial dysfunction is an important characteristic of oxidative stress-induced senescence. Autophagy.

[45] Liu X., Wu Y., Zhou D., Xie Y., Zhou Y., Lu Y., Yang R., Liu S. (2020). N-linoleyltyrosine protects PC12 cells against oxidative damage via autophagy: Possible involvement of CB1 receptor regulation. Int. J. Mol. Med..

[46] Feng Y., Jiang C., Yang F., Chen Z., Li Z. (2020). Apocynum venetum leaf extract protects against H_2_O_2_-induced oxidative stress by increasing autophagy in PC12 cells. Biomed. Rep..

[47] Corti O., Blomgren K., Poletti A., Beart P.M. (2020). Autophagy in neurodegeneration: New insights underpinning therapy for neurological diseases. J. Neurochem..

[48] Zhang Z., Ji Y. (2020). Nanostructured manganese dioxide for anticancer applications: Preparation, diagnosis, and therapy. Nanoscale.

[49] Zhang Y., Chen L., Sun R., Lv R., Du T., Li Y., Zhang X., Sheng R., Qi Y. (2022). Multienzymatic antioxidant activity of manganese-based nanoparticles for protection against oxidative cell damage. ACS Biomater. Sci. Eng..

[50] Pouokam E., Rehn M. (2009). Effects of H_2_O_2_ at rat myenteric neurones in culture. Eur. J. Pharmacol..

[51] Huang Y., Ruan Y., Ma Y., Chen D., Zhang T., Fan S., Lin W., Huang Y., Lu H., Xu J.-F. (2023). Immunomodulatory activity of manganese dioxide nanoparticles: Promising for novel vaccines and immunotherapeutics. Front. Immunol..

[52] Gavin C.E., Gunter K.K., Gunter T.E. (1999). Manganese and calcium transport in mitochondria: Implications for manganese toxicity. Neurotoxicology.

[53] Kma L., Baruah T.J. (2022). The interplay of ROS and the PI3K/Akt pathway in autophagy regulation. Biotechnol. Appl. Biochem..

[54] Qiao C.M., Sun M.F., Jia X.B., Shi Y., Zhang B.-P., Zhou Z.-L., Zhao L.-P., Cui C., Shen Y.-Q. (2020). Sodium butyrate causes alpha-synuclein degradation by an Atg5-dependent and PI3K/Akt/mTOR-related autophagy pathway. Exp. Cell Res..

[55] Chu Q., Yu L., Zheng Z., Chen M., Hua Z., Hang M., Li Y., Li X., Liu Y., Yang Y. (2019). Apios americana Medik flowers extract protects PC12 cells against H_2_O_2_ induced neurotoxicity via regulating autophagy. Food Chem. Toxicol..

[56] Qi M., Wang Q., Sun R., Cheng Z., Li M., Fan X., Bai F., Yu J. (2025). Astragaloside IV alleviates H_2_O_2_-induced mitochondrial dysfunction and inhibits mitophagy via PI3K/AKT/mTOR pathway. Cardiovasc. Ther..

[57] Han Q., Zhang W., Lu C., Wu J., An S., Zhang S. (2020). Repression of Kisspeptin1 weakens hydrogen peroxide-caused injury in HTR8 cells via adjusting PI3K/AKT/mTOR pathway. J. Biochem. Mol. Toxicol..

[58] Moon J.M., Lee S.W., Jang Y.S., Lee S.-A., Jung S.-H., Kim S.-K., Park B.-K., Park Y.-S., Kim B.-S., Yang M.-S. (2025). Gossypin induces apoptosis and autophagy via the MAPK/JNK pathway in HT-29 human colorectal cancer cells. Int. J. Mol. Med..

