# Supplementary material for: Manganese Dioxide Nanoparticles Protect PC12 Cells Against H2O2-Induced Oxidative Stress Injury by Regulating PI3K/Akt-Mediated Autophagy
Source: Pharmaceutics. 2026 Sep 15;18(9):1156. doi: 10.3390/pharmaceutics18091156 (PMC13609983; doi:10.3390/pharmaceutics18091156)
Supplement: Supplementary file 1 [file pharmaceutics-18-01156-s001.zip › pharmaceutics-4479502-supplementary.pdf]

pharmaceutics

MDPI

Article

Manganese Dioxide Nanoparticles Protect PC12 Cells Against H_2O_2-Induced Oxidative Stress Injury by Regulating PI3K/Akt-Mediated Autophagy

Weijian Zeng [1,†], Duanyang Zhou [2,†], Tianlong Wang [1], Zhan-Lu Ma-Högemeier [1], Song Cai [2], Bingfeng Liu [3], Chao Song [4], Ling Guo [4], Rihong Zhai [2], Xun Song [1], Zhendan He [1] and Yun Dong [1,*]

[1] College of Pharmacy, Shenzhen Technology University, Shenzhen 518118, China; zwj040820@163.com (W.Z.); wangtianlong@sztu.edu.cn (T.W.); mazhanlu@sztu.edu.cn (Z.-L.M.-H.); songxun@sztu.edu.cn (X.S.); hezhendan@sztu.edu.cn (Z.H.)

[2] School of Pharmaceutical Sciences, Health Science Center, Shenzhen University, Shenzhen 518055, China; 2070245005@email.szu.edu.cn (D.Z.); caisong@szu.edu.cn (S.C.); rzhai@szu.edu.cn (R.Z.)

[3] The Brain Cognition and Brain Disease Institute, Shenzhen Institute of Advanced Technology, Chinese Academy of Sciences, Shenzhen 518055, China; bf.liu@siat.ac.cn

[4] Anning First People's Hospital Affiliated to Kunming University of Science and Technology, Kunming 650302, China; chaoge6870@163.com (C.S.); h0726136@kust.edu.cn (L.G.)

* Correspondence: dongyun@sztu.edu.cn

† These authors contributed equally to this work.

Electrophysiological Determination of Sodium Current

The extracellular recording solution contained 140 mM NaCl, 30 mM tetraethylammonium chloride, 10 mM D-glucose, 3 mM KCl, 1 mM $CaCl_2$, 0.5 mM $CdCl_2$, 1 mM $MgCl_2$, and 10 mM HEPES, and the pH of the solution was adjusted to 7.3 using NaOH with an osmolarity of 310-315 mOsm/kg. The intracellular recording solution contained 140 mM CsF, 10 mM NaCl, 15 mM HEPES and 1.1 mM Cs-EGTA, and the pH of the solution were adjusted to 7.3 using CsOH. After all treatments, PC12 cells were recorded using current-voltage (I-V) protocols. The I-V protocols were as follows: PC12 cells were clamped at -80 mV, and the cells were depolarized in 200 ms steps in the range of -70 mV to +60 mV (+5 mV increments). This operation results in a current density so that the activation of sodium channels was generated between 0 mV to10 mV. This process occurred between 0 mV and 10 mV and could be analyzed as a function of voltage, as inferred from the peak current density (normalized to the cell capacitance (picofarad, pF). Whole-cell recording was performed using a HEKA EPC-USB 10 (HEKA Instruments). The pipettes used had a resistance of 2-4 MΩ. Data were obtained by Patchmaster (HEKA) and analyzed with Fitmaster (HEKA). Capacitive artifacts were fully compensated, and series resistance was compensated by approximate 60%. All experiments were performed at room temperature.

Academic Editors: Christian Celia and Nadia Araci Bou-Chacra

Received: 20 July 2026
Revised: 16 August 2026
Accepted: 3 September 2026
Published: 15 September 2026

Copyright: © 2026 by the authors. Licensee MDPI, Basel, Switzerland. This article is an open access article distributed under the terms and conditions of the Creative Commons Attribution (CC BY) license.

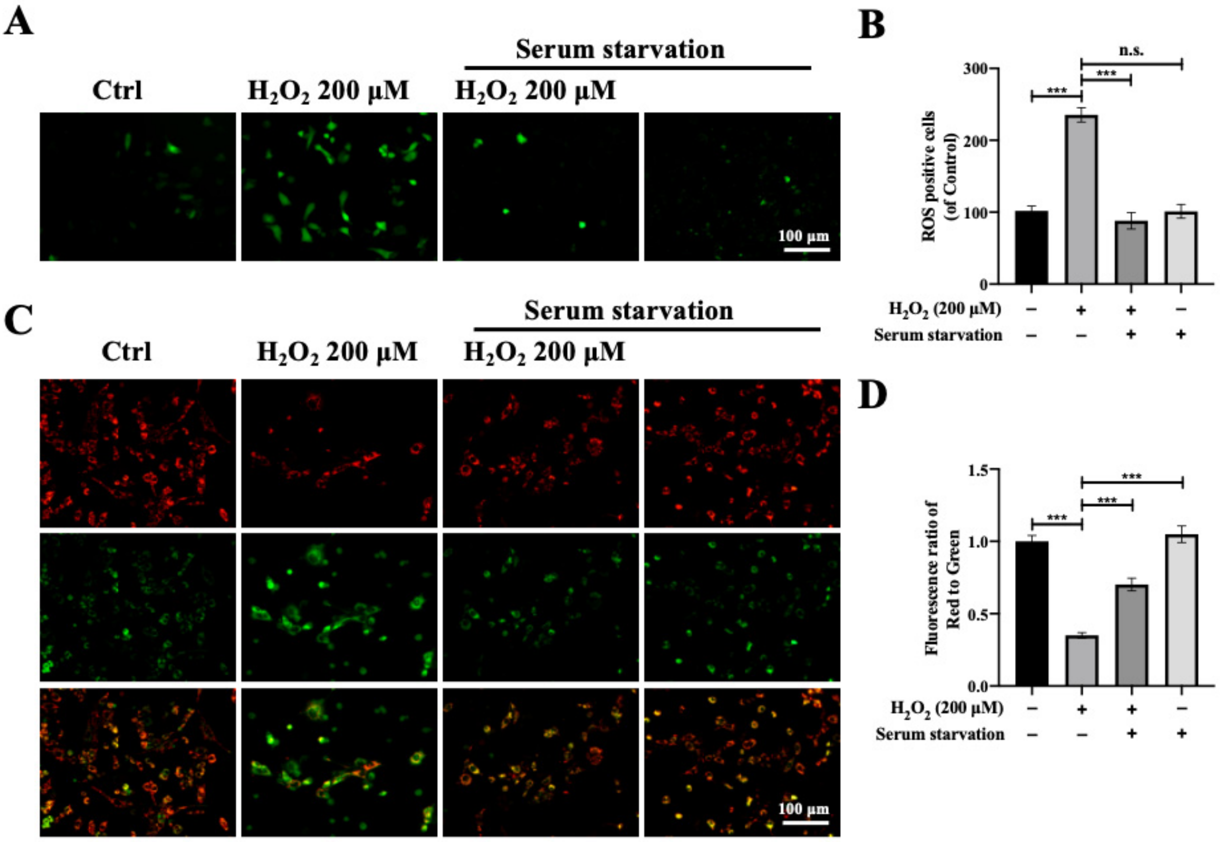

Figure S1. Serum starvation reduces ROS production and mitochondrial dysfunction. (**A**) Treated PC12 cells were stained with DCFH-DA, and (**B**) fluorescent density mean of ROS level was calibrated. Scale bar = 100 μm. (**C**) Treated PC12 cells were stained with JC-1 kit. (**D**) Ratios of JC-1 green/red fluorescence were calibrated. Scale bar = 100 μm. All data in bar charts represent mean ± SEM from at least three independent experiments. *** $p < 0.001$ versus the H_2O_2 group or the control group (Ctrl). n.s, no significance.

https://doi.org/10.3390/pharmaceutics18091156

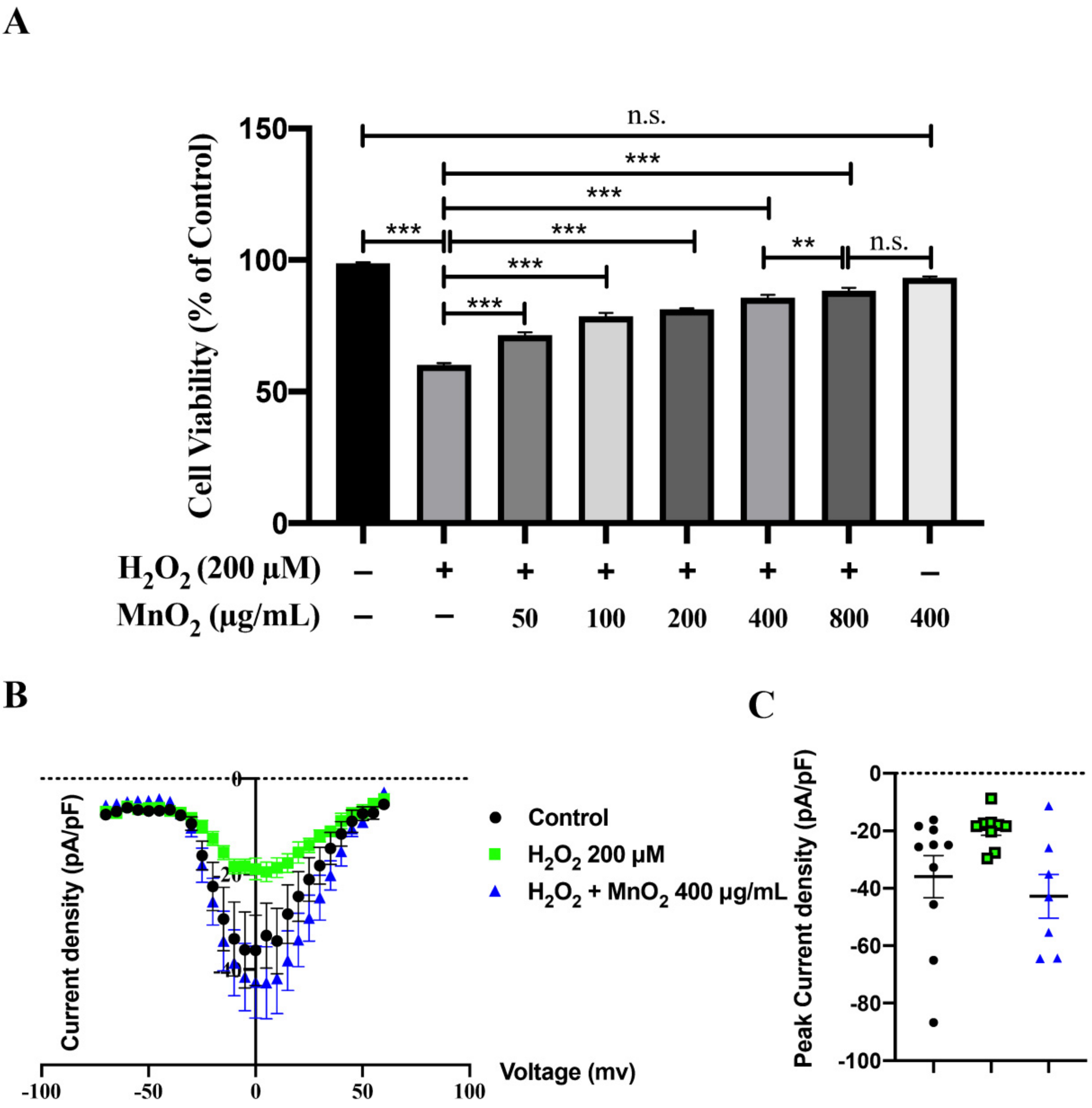

Figure S2. BSA-MnO2 prevents H2O2-induced oxidative damage in PC12 cells. (A) Cell viability was detected. (B) Statistics for the peak Na+ current (INa). (C) Current-Voltage (I-V) relationship for the INa recorded. All data in bar charts represent mean ± SEM from at least three independent experiments. ****p* < 0.001 versus the H_2O_2 group or the control group (Ctrl). n.s, no significance.

https://doi.org/10.3390/pharmaceutics18091156

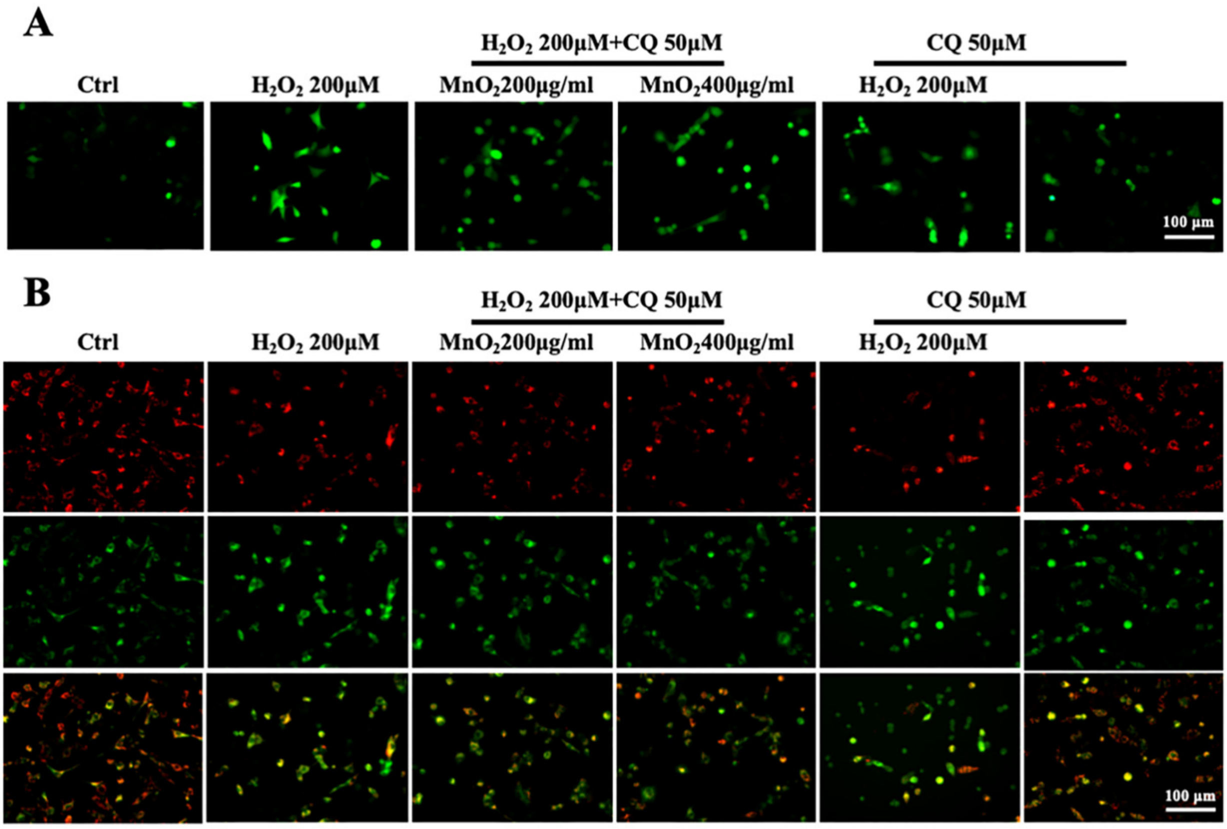

Figure S3. The recovered mitochondrial membrane potential and reduced ROS generations mediated by BSA-MnO_2 against H_2O_2 were abolished by CQ. (A) Treated PC12 cells were stained with DCFH-DA Scale bar = 100 μm. (B) Treated PC12 cells were stained with JC-1 kit Scale bar = 100 μm. All data in bar charts represent mean ± SEM from at least three independent experiments.

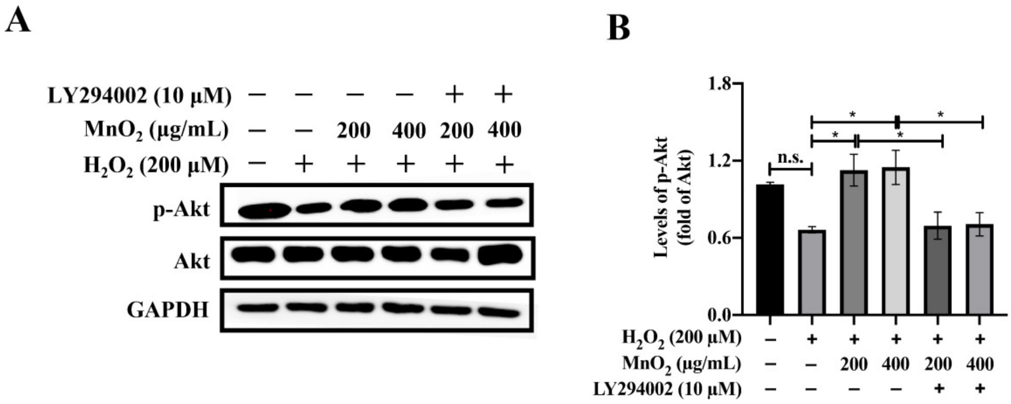

Figure S4. Expression of p-Akt up-regulated by BSA-MnO_2 is decreased by Ly294002. (A) The protein expression of p-Akt and Akt were determined by western blotting. Expression of GAPDH was served as loading control. (B) Quantitation of p-Akt expression was calibrated. All data in bar charts represent mean ± SEM from at least three independent experiments. $^*p < 0.05$ versus the H_2O_2 group (Ctrl). n.s., no significance.

https://doi.org/10.3390/pharmaceutics18091156

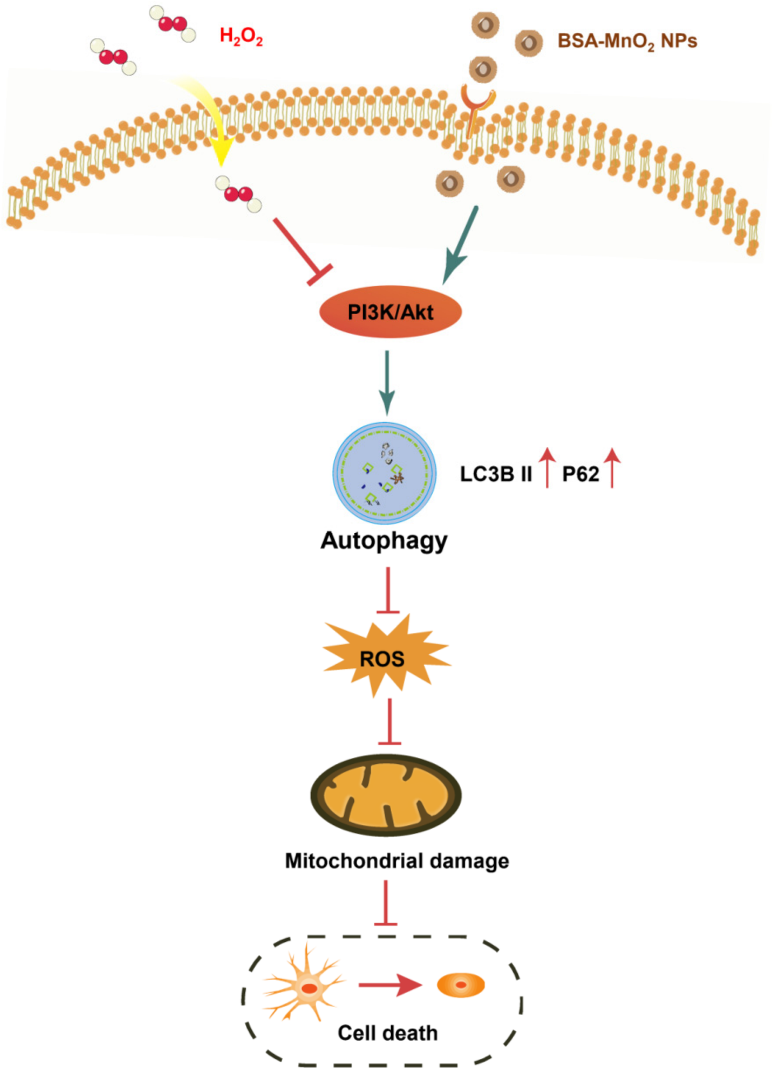

Figure S5. The schematic diagram of the neuroprotection of BSA-MnO_2 against H_2O_2-induced oxidative damage in PC12 cells.

https://doi.org/10.3390/pharmaceutics18091156
